# Human cerebral organoids with microglia and vasculature model glioma stem cell interactions and radiotherapy response

**DOI:** 10.1016/j.crmeth.2026.101425

**Published:** 2026-05-05

**Authors:** Jérémy Raguin, Noa Legrand, Thierry Kortulewski, Oriane Bergiers, Christine Granotier-Beckers, Laure Chatrousse, Alexandra Benchoua, Laurent R. Gauthier, François D. Boussin, Marc-André Mouthon

**Affiliations:** 1 Université Paris Cité, Inserm, CEA, Stabilité Génétique Cellules Souches et Radiations, LRP/iRCM/IBFJ, 92265 Fontenay-aux-Roses, France; 2 Université Paris-Saclay, Inserm, CEA, Stabilité Génétique Cellules Souches et Radiations, LRP/iRCM/IBFJ, 92265 Fontenay-aux-Roses, France; 3 IStem, CECS, AFM, Neuroplasticity and Therapeutics, 91100 Corbeil-Essonne, France

**Keywords:** iPSC, cerebral organoids, microglia, vascularized, endothelial cells, glioma, radiotherapy, glioblastoma, brain cancer, precision medicine

## Abstract

Most human brain organoid models derived from induced pluripotent stem cells (iPSCs) lack vascular and/or immune components, despite their critical roles in maintaining brain homeostasis and contributing to pathophysiological processes. We established a method for generating vascularized complex cerebral organoids (CCOs) containing microglial cells (brain-resident macrophages) by incorporating bipotent hematopoietic/endothelial progenitors derived from the same iPSC lines. This approach led to the formation of extensive vascular-like structures with blood-brain barrier characteristics, which were perfused upon transplantation into immunodeficient mice. Additionally, microglial cells exhibiting typical phenotypes also developed within the CCOs. By co-culturing CCOs with glioma stem cells, we demonstrated that this model recapitulates the tumor niche of glioblastoma, showing vascular co-option, reprogramming of microglia into tumor-associated macrophages, and recurrence after radiotherapy. In conclusion, our vascularized, immunocompetent CCO model provides a platform to study brain development, glioma pathogenesis, and therapies.

## Highlights

• Generation of vascularized cerebral organoids with functional microglia
• Organoids form perfusable vasculature with BBB features
• Invasion of glioma stem cells into cerebral organoids mimics the tumor microenvironment
• Model can potentially enable study of brain development, glioma biology, and therapy

## Motivation

Understanding of glioblastoma resistance and recurrence is hindered by the lack of human models that faithfully recapitulate the tumor microenvironment. Vascular and immune components, particularly tumor-associated macrophages, play critical roles in tumor progression and therapy failure, yet most brain organoids lack these features. Developing vascularized and immunocompetent cerebral organoids is, therefore, essential to better model GBM biology and to identify more effective therapeutic strategies.

## Graphical abstract

**Figure:**
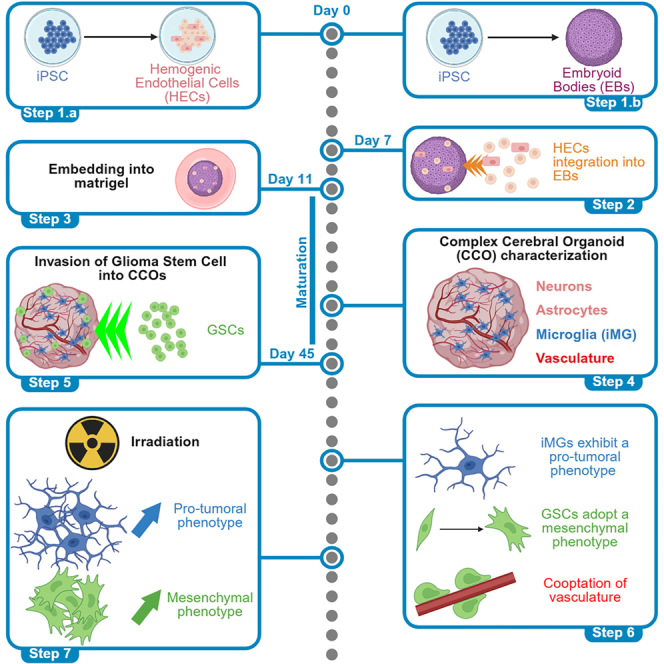

## Teaser

Raguin et al. develop a method to generate vascularized, immunocompetent cerebral organoids by incorporating iPSC-derived hematoendothelial progenitors. These complex organoids form perfusable vascular-like networks with blood-brain barrier features and functional microglia. When co-cultured with glioma stem cells, they recapitulate key aspects of the glioblastoma niche.

## Introduction

Glioblastoma (GBM) is a brain tumor that is fatal despite aggressive treatment combining surgery, radiotherapy, and chemotherapy. Glioma stem cells (GSCs), a subpopulation of tumor cells with stem cell properties, are thought to play a major role in tumor relapse due to their resistance to treatment and invasive capacity.^1^^,^^2^

The innate immune and vascular systems within the tumor microenvironment also play a key role in GBM’s resistance to treatment^3^ and undergo significant alterations following radiotherapy.^4^ Indeed, tumor-associated macrophages (TAMs) represent the majority of non-tumor cells in GBM, and their abundance correlates with disease severity, poor survival, and recurrence.^5^^,^^6^ TAMs create immunosuppressive and pro-angiogenic conditions through the production of cytokines, promoting the proliferation and survival of GBM cells by synthesizing growth factors.^3^ TAMs represent a heterogeneous population originating either from microglial cells (resident macrophages) or from circulating monocytes and/or bone marrow-derived cells.^7^ The nature of TAMs depends, in part, on the genetic status of the tumor. In GBMs (isocitrate dehydrogenase (*IDH*) gene wild-type tumors), TAMs are predominantly derived from monocytes, whereas in *IDH*-mutated astrocytomas, they are more associated with microglial cells.^8^ Vessel co-option and vascularization are among the strategies employed by some GBM cells to further invade the brain and regulate their proliferation and survival.^9^ In the peri-necrotic regions of GBM, an abundance of destabilized microvessels polarizes macrophages toward a hypoxic state, which, in turn, increases vascular hyperpermeability, disrupting the efficacy of antitumor drugs.^10^

Altogether, these mechanisms contribute to the failure of various therapeutic approaches for GBM, but the exact roles of the GBM microenvironment in tumor development and relapse after treatments remain incompletely understood. Their elucidation requires study models that replicate its complexity in humans, which are currently not available.

The recent development of human brain organoids derived from human induced pluripotent stem cells (iPSCs) allows for the modeling of brain biology and GBM pathophysiology.^11^ Indeed, using 3D co-culture systems of human GSCs and brain organoids enables the identification of invasion signatures similar to those observed in surgical samples from GBM patients.^12^^,^^13^ However, 3D co-culture models combining GBM cells and brain organoids have certain limitations, particularly the absence of vascularization and immune cells. On one hand, vascularized organoids have been obtained using various sources such as endothelial cells (ECs) and genetically modified iPSCs^14^ or after their transplantation into mice.^15^ On the other hand, microglia-containing cerebral organoids (COs) have been generated (reviewed by Zhang et al.^16^) with functional microglial cells^17^ that closely resemble their *in vivo* counterparts.^18^ Recently, COs containing both vascular systems and microglial cells have also been created by assembling vascular organoids and COs.^19^

Brain vasculogenesis, the *de novo* formation of blood vessels, begins during embryoid development with the emergence of hemogenic ECs (HECs) in the mammalian extraembryonic yolk sac.^20^ Brain-resident macrophages, or microglia, also originate from this wave of HECs in the yolk sac and colonize the brain during early embryoid development.^21^ The emergence of these HECs, which are of mesodermal origin, has been modeled using iPSCs.^22^ Indeed, several research groups have developed strategies to derive HECs, inspired by human embryoid development.^23^ HECs display a dual endothelial-hematopoietic phenotype characterized by the expression of VEGFR2/KDR/CD309 and the endothelial-specific junctional protein vascular endothelial cadherin (Cdh5/CD144).^24^ HECs also have the capacity to differentiate into various mesodermal cell types, including pericytes.^24^

In this study, we developed a method to create vascularized COs containing microglial cells from iPSCs derived from healthy donors, with the aim of establishing an *in vitro* model for GBM. Our strategy was to recapitulate the stages of embryoid brain development, including the colonization of COs by HECs. By incorporating HECs during the early stages of CO formation, we generated complex cerebral organoids (CCOs) containing both vascular structures and microglia. Finally, the invasion of GSCs within these vascularized, immunocompetent COs served as a model to recapitulate the GBM tumor niche.

## Results

### Integration of HECs favored CO maturation

We designed a stepwise protocol by separately inducing mesodermal differentiation into HECs and neuroectodermal differentiation from the same iPSC lines, then combining them to generate CCOs. We stimulated the differentiation of two iPSC lines (PDF01 and GM25256) into HECs by using a recently developed protocol with some modifications.^23^ Hematopoietic-endothelial differentiation of iPSCs was confirmed by the expression of markers—determined through fluorescence-activated cell sorting (FACS) and quantitative reverse-transcription PCR (RT-qPCR) for CD309/KDR and CD144/Cdh5—and by FACS for CD31 and CD146—the mesenchymal cell/EC markers (Figure S1). RUNX1, a transcription factor involved in hematopoiesis, was also expressed at the mRNA level (Figure S1).

Meanwhile, embryoid bodies were formed from the same two iPSC lines to derive COs,^25^ which were then co-cultured with HECs on seventh day in the presence of VEGFA, IL-34, and GM-CSF to support their differentiation into vascular and microglial cells.

Given the presence of lineage-directing growth factors and the incorporation of HECs into the COs, we examined whether neuroectodermal differentiation remained unaffected. CCOs were collected between days 49 and 113 after initiation and processed for immunostaining, either on thin slices or clarified thick slices, followed by 3D confocal microscopy. The CCOs contained neural rosettes composed of SOX2-positive neural progenitors and SOX9-positive gliogenic progenitors (Figures S2A and S2B). However, neural rosettes appeared sparsely distributed, suggesting the progressive differentiation of neural progenitors. Neural precursors expressing doublecortin (DCX) were abundant and widely distributed within the CCOs (Figure S2C). Differentiation into neurons was confirmed by the presence of MAP-2 and β3-tubulin immunostaining, which were widely present throughout the CCOs derived from both iPSC lines (Figures S2D, S2E, and S2E′). The presence of numerous astrocytes was confirmed by S100β/GFAP double-positive cells (Figures S3A, S3B, and S3B′). Notably, numerous astrocytes were observed in CCOs at 81 days *in vitro* (DIV), a time point when they were still absent from classical organoids, which showed only sparse astrocytes even at 126 DIV (Figures S3C and S3D).

Altogether, these findings indicated that the incorporation of HECs did not hinder neuronal differentiation, and even appeared to accelerate astrocyte emergence, suggesting that it promoted CO maturation.

### Pseudo brain vascularization in CCOs

**Figure 1:**
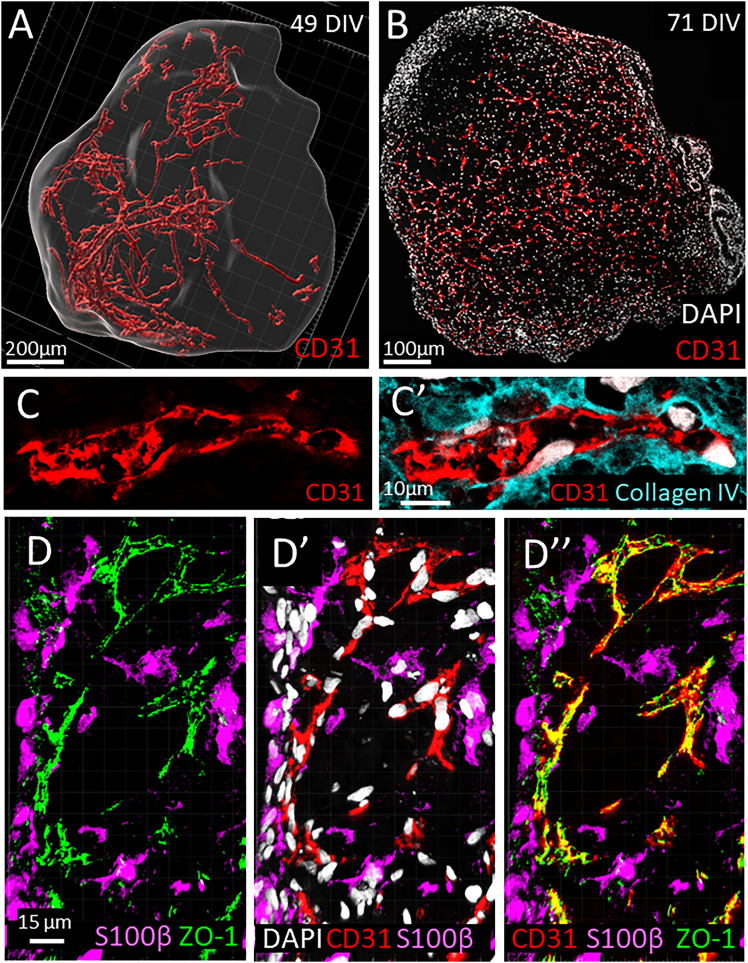
Vascular-like structures developed in CCOs (A–D) Immunostaining for CD31 performed on 500 μm sections (A) and 5 μm thin sections (B–D). 3D modeling of CD31 is shown in (A). Enlarged views of the organoids derived from the PDF01 line (A) and GM25256 line (B) are shown. (C) and (C′) show enlarged views of (B), together with CD31 and collagen IV immunostaining. Multiplex immunostaining for endothelial cells (CD31), astrocytes (S100β), and BBB-specific tight junction (ZO-1) was performed at DIV71 and taken at 63×. (D)–(D″) show that CD31-positive vascular-like structures are in close contact with S100β cells and exhibit ZO-1 tight junctions. CCOs were derived from PDF01 (A) and GM25256 (B–D) iPSC lines.

Fifty days after initiation, numerous tubule-like structures positive for CD31, a specific EC marker, had extensively developed within the CCOs derived from both iPSC lines (Figures 1A and 1B). These vascular structures occupied a significant portion of the organoids, including the inner region (Figures S4A and S4A′), but excluding the nucleus-dense areas, which were mainly composed of β3-positive neurons (Figures S4B and S4B′). These vascular-like networks were intermingled with neuronal terminals (Figure S4B). They were observed in CCOs initiated from both iPSC lines tested and remained prominent at later time points (up to 126 days). Additionally, immunostaining for collagen IV was detected adjacent to CD31, resembling the basal lamina of blood vessels (Figures 1C and 1C′). To confirm that the basal lamina was synthesized in association with the vascular structures, rather than being solely derived from the Matrigel, we performed immunostaining using a human-specific antibody against laminin α5. Indeed, laminin α5 signal was closely apposed to collagen IV, outlining vascular-like structures, whereas it was absent in conventional COs (Figures S5A and S5B). To determine whether these vessel-like structures mimic the blood-brain barrier (BBB), we performed multiplex immunohistological analyses on thin CCO slices. ZO-1, a marker of tight junctions in brain vessels, was found to be expressed on CD31^+^ ECs in the CCOs (Figure 1D). In addition, the detection of S100β^+^ cells adjacent to vascular structures was consistent with the presence of astrocytes, a component of the BBB (Figure 1D).

**Figure 2:**
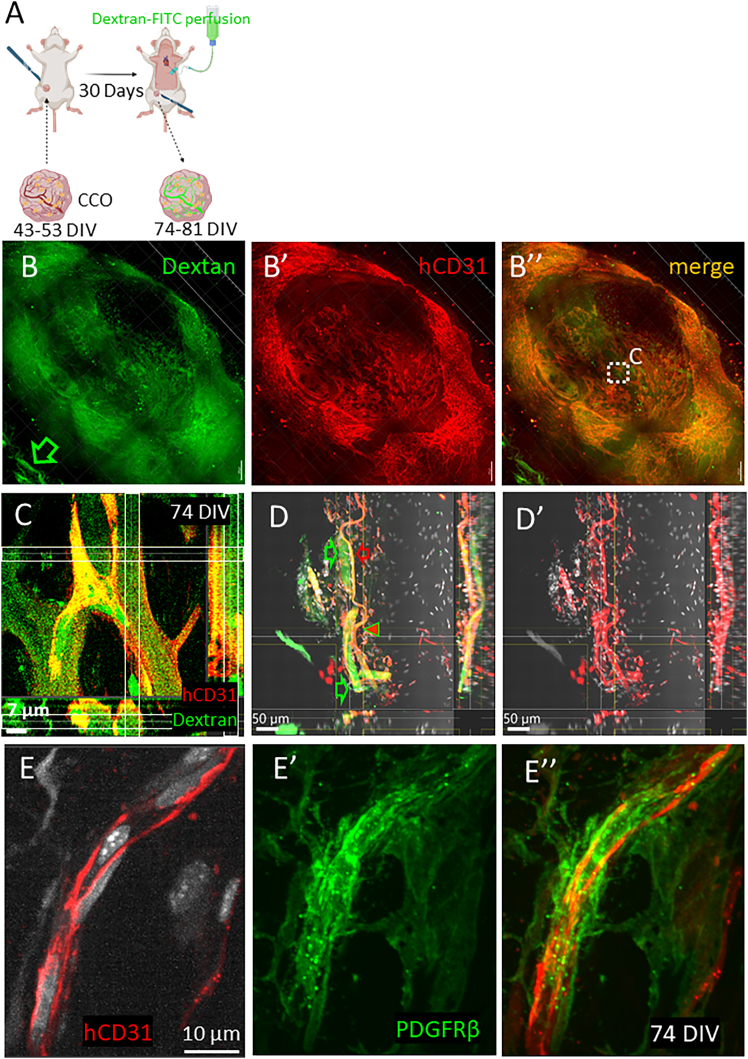
Vascular-like structures in CCOs are perfused after xenotransplantation and are covered by pericytes (A) CCOs were xenotransplanted subcutaneously into the mouse limb. One month after transplantation, intracardiac perfusion with FITC-dextran was performed to label perfused blood vessels. (B) FITC-dextran-positive vessels were observed on 500-μm thick sections both within the CCO and in adjacent mouse vessels (green arrow). The specificity of the human anti-CD31 immunostaining was confirmed by the absence of signal in murine adjacent vessels. (B′) Human CD31 immunostaining on 500-μm thick CCO sections. (C) A magnified 3D reconstruction of CD31 immunostaining reveals a vessel lumen perfused with FITC-dextran within the CCO. (D and D′) Mouse vessels are visualized by FITC-dextran (green arrows), while human CCO vessels are marked by CD31 (red arrow). A vascular anastomosis between host and graft vessels is visible (bicolored arrowhead). (E–E″) Vascular structures are covered by PDGFRβ-positive pericytes. CCOs were derived from GM25256 iPSCs. Xenotransplantations were performed in six mice across two independent experiments.

To further examine the potential functionality of these vessel-like structures, CCOs were transplanted into immunodeficient NOD-SCID mice. One month post-transplantation, dextran-FITC was infused intracardially into the mice’s vascular system, enabling visualization of vessel-like structures within the CCOs (Figures 2A and 2B). Notably, these dextran-FITC-perfused vessel-like structures in CCOs were also immunostained with a specific anti-human CD31 antibody (Figures 2B–2D), in contrast to FITC-perfused murine vessels adjacent to CCOs (Figure 2B). Figures 7D and 7D′ further show the anastomosis between mouse and human CCO-derived vessels. Moreover, human CD31^+^ cells had typical elongated nuclei and were covered by PDGFRβ-positive pericytes (Figures 2E and E″).

Overall, our data show that vascular-like structures in CCOs contain components of the neurovascular unit and exhibit BBB-like features.

### Microglia-like cells developed in CCOs

**Figure 3:**
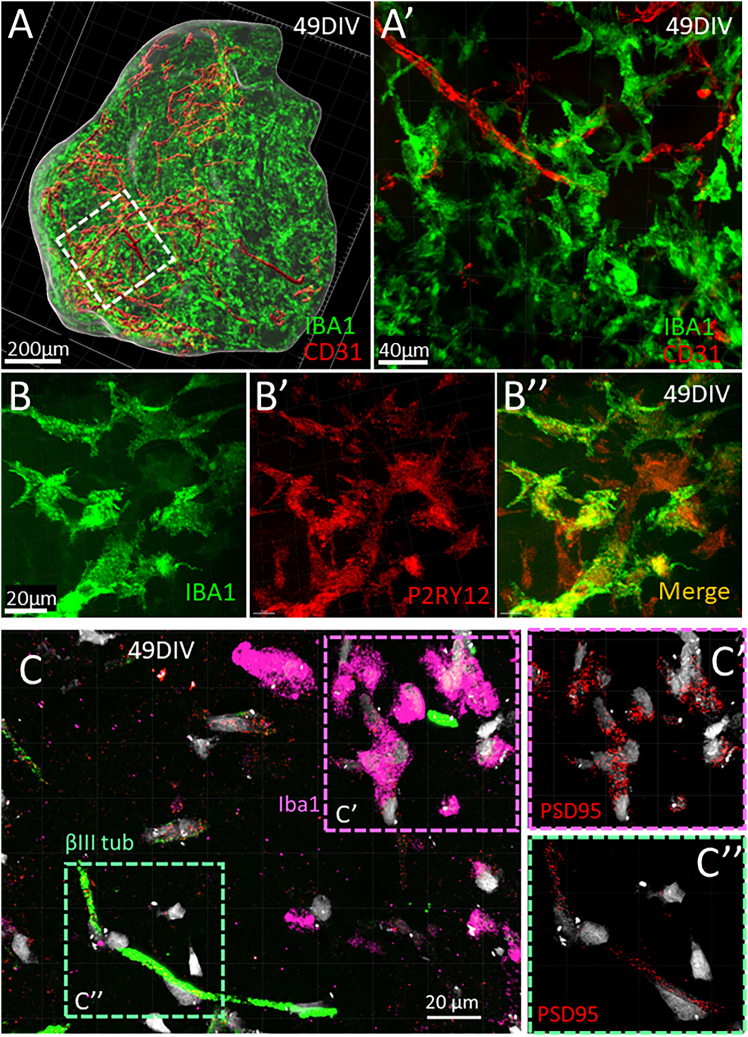
Microglial-like cells developed in CCOs and internalized the post-synaptic protein PSD95 (A and B) Representative immunostaining images for CD31 and IBA1 on 500 μm section of the CCO. Enlarged view is shown in A′. (B–B″) Almost all Iba1-positive cells co-expressed the microglial marker P2RY12. (C–C″) PSD95 immunostaining on a 5-μm thick section observed in βIII-tubulin^+^ neurons (C″), and Iba1-expressing cells showed internalization of the neuronal PSD95. The outline of Iba1^+^ cell is shown in Cʹ. CCOs were derived from the PDF01 iPSC line (A and B) and GM25256 iPSC line (C).

**Figure 4:**
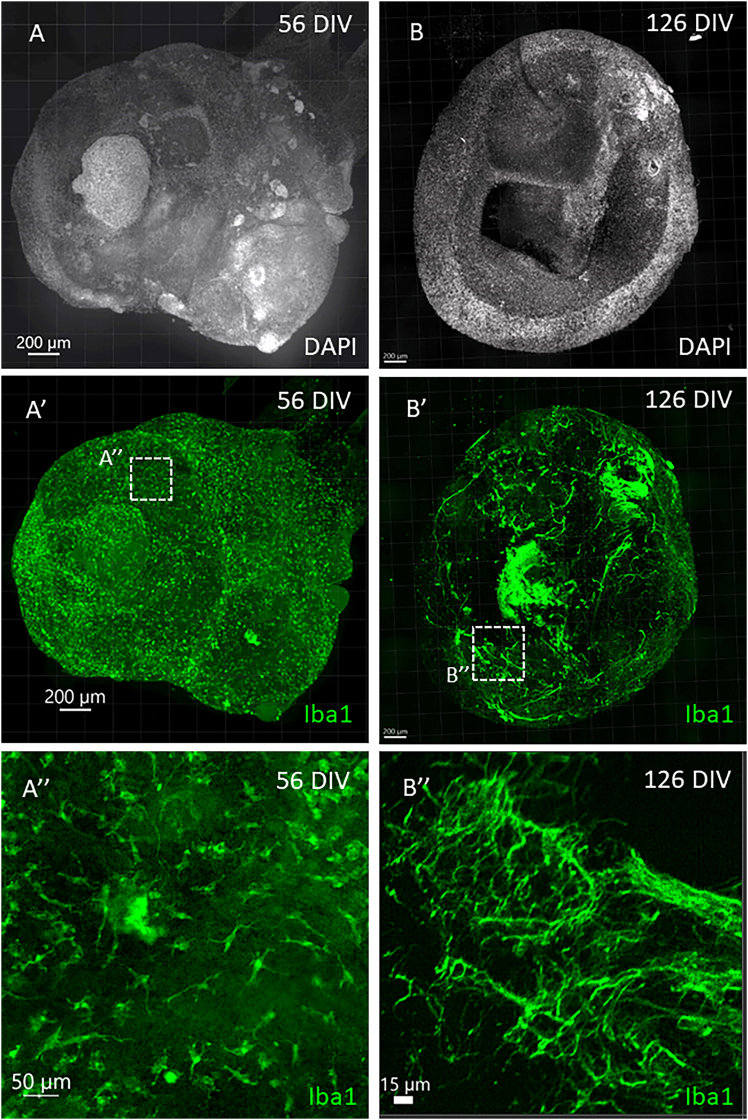
Iba1^+^ cells exhibited increasingly elongated processes and greater morphological complexity over time (A and B) Iba1 immunostaining on 400-μm thick CCO section at 56 DIV (A–A″) and 126 DIV (B–B″). CCOs were derived from the GM25256 iPSC line. A magnified view of Iba1 immunostaining is shown in (A″) and (B″).

Next, we analyzed whether the early incorporation of HECs into embryoid bodies effectively led to the generation of microglial cells in the CCOs. Fifty days after initiation, numerous Iba1-positive cells, a pan-macrophage/microglia marker, were widely observed, extensively infiltrating the CCOs derived from both iPSC lines (Figures 3A and 4). Virtually, all Iba1-positive cells were also positive for P2RY12, a specific microglial marker (Figure 3B–B″). Additionally, while Iba1^+^ cells exhibited an immature morphology at the early time points (56 DIV), they developed more mature microglial features at later stages (126 DIV), characterized by increased process length and morphological complexity (Figure 4B″).

Microglial cells play crucial roles in synaptic pruning and promoting brain maturation. To demonstrate that Iba1^+^ cells have this function in CCOs, we examined their ability to engulf synapses by double staining for postsynaptic density protein 95 (PSD95) and Iba1. Remarkably, Iba1^+^ cells exhibited numerous PSD95 puncta distributed throughout their cytoplasm, indicating synaptic engulfment (Figure 3C).

### Quantification of vascular and microglial cells in CCOs

**Figure 5:**
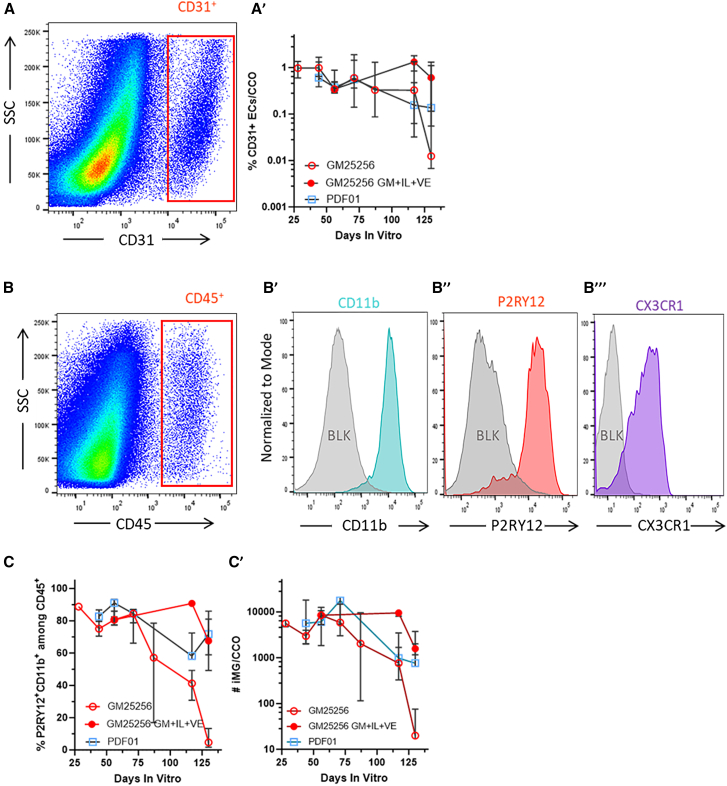
Flow cytometry-based quantification of key cellular populations in CCOs as a function of time and the iPSC line (A) Endothelial cells were defined as CD31^+^ cells, and quantification with iPSC lines are shown (A′). (B) Microglial-like cells were contained in the CD45^+^ population expressing CD11b (B′), P2RY12 (B″), and CX3CR1 (B‴). (C) For both iPSC lines, the great majority of CD45^+^ co-expressed CD11b and P2RY12, but this proportion dropped at a later time point when exogenous factors were removed. (C′) The number of CD45^+^CD11b^+^P2RY12^+^ cells (iMGs) was maintained over time in the presence of GM-CSF, IL-34, and VEGF (GM + IL + VE), but decreased at later time points in CCOs derived from both iPSC lines when exogenous factors were withdrawn. CCOs were derived from PDF01 and GM25256 iPSC lines. Data are presented as median with interquartile range; each data point represents 2–16 individual CCOs.

To quantify vascular- and microglia-like populations, we performed flow cytometry on single-cell suspensions generated by the enzymatic dissociation of CCOs. ECs, identified by CD31 expression, were detected in CCOs but not in conventional COs. The frequency of CD31^+^ ECs in CCOs did not differ between the iPSC lines (Figure 5A). Microglial cells express the hematopoietic marker CD45, and significant levels of CD45^+^ cells were found in CCOs while very few, if any, CD45^+^ cells were detected in conventional COs (Figures S6D and S6E). In addition, CD45^+^ cells were positive for the myeloid marker CD11b and the specific markers of resident microglia, i.e., P2RY12 and CX3CR1 (Figures 5B and S6Dʹ–D″). Interestingly, the great majority of CD45^+^ cells in CCOs were P2RY12^+^CD11b^+^ microglial-like cells without any difference according to iPSC lines (Figures 5C and 5C′). The maximum number of CD45^+^CD11b^+^P2RY12^+^ cells, i.e., microglia, was observed around ten weeks after initiation of CCOs (Figure 5C). Although RT-qPCR analyses showed endogenous expression of VEGFA, IL-34, and CX3CL1—growth factors involved in endothelial and microglial development/homeostasis (Figure S7A)—the withdrawal of exogenous VEGF, GM-CSF, and IL-34 after day 30 led to a marked reduction in CD31^+^ ECs and CD45^+^CD11b^+^ P2RY12^+^ microglia-like cells (Figures 5A and 5C′).

We further confirmed the expression of macrophage/microglial genes in sorted CD45^+^ cells. The hematopoietic transcription factors SPI1 and RUNX1 were highly expressed in CD45^+^ cells compared with parental iPSCs and differentiated HECs (Figure S7B). Additionally, CSF1R and AIF1 were also enriched in CD45^+^ cells (Figure S7B). Other markers, including P2RY12, TMEM119, PLIN2, and CX3CR1, showed high enrichment in CD45^+^ cells compared with iPSCs and differentiated HECs (Figure S7B). Therefore, these CD45^+^/Iba1^+^ cells are referred to as induced microglia (iMG) hereafter.

Overall, our data demonstrated that both iPSC lines generated CCOs containing functional iMG and BBB-like vascular structures.

### Co-culture of GSCs with CCOs recreated heterogeneity of tumor microenvironment

We next investigated whether our organoid model could efficiently recapitulate the GBM tumor niche. GBM tumor niches consist of tumor cells, including GSCs, non-neoplastic cells such as neural and vascular cells, and TAMs. To this end, we performed 3D co-cultures of CCOs with the GSC lines derived from adult patients with wild-type *IDH* GBM (TG16) or *IDH*-mutated grade IV astrocytoma (TG20) (Figure S8). Both GSC lines exhibited high expression of the stem cell marker SOX2, as revealed by FACS, with TG16 showing elevated levels of CD44 (Figure S8B), consistent with its mesenchymal phenotype.^26^ Additionally, RT-qPCR showed that TG16 GSCs expressed higher levels of chemokines, cytokines, and immune checkpoints than TG20 GSCs (Figures S8B and S8C). Following xenotransplantation into nude mice, both GSC lines generated tumors and induced host death, which occurred earlier with TG16 GSCs than with TG20 GSCs (Figures S8D and S8E). *In situ* analyses further revealed that GSC-derived tumors displayed vascular abnormalities and extensive infiltration of Iba1^+^ macrophages/microglial cells (Figures S8F and S8G). FACS analyses of dissected striatum showed a higher abundance of CD45^++^CD11b^+^ cells, corresponding to monocyte-derived TAMs, in tumor-bearing brains than in controls (Figures S8H and S8H′). The abundance of CD206^+^ macrophages, which are associated with an immunosuppressive phenotype, was also higher in tumor-bearing mice (Figure S8H″). Additionally, sorted CD45^+^ cells, encompassing both macrophages and microglia, exhibited elevated expression of key genes (*MSR1*, *SPP1*, and *TGF-β1*), indicating their transition to an immunosuppressive TAM phenotype (Figure S8I) consistently with the expression of cytokines and immunomodulators by GSCs (Figure S8C).

**Figure 6:**
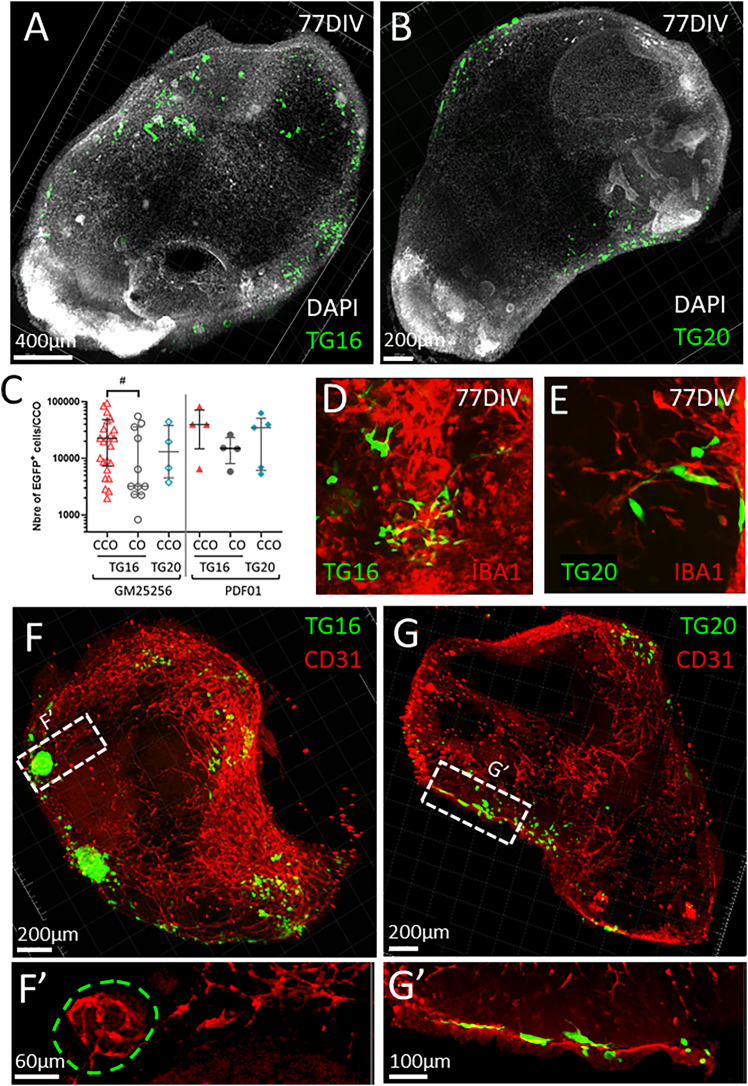
Co-cultures of GSC-derived lines with CCOs mimicked the tumor microenvironment (A and B) TG16-GSCs and (B) TG20-GSCs were co-cultured with CCO for 4 weeks and were immunodetected for EGFP on 500 μm sections. (C) Quantification by FACS of EGFP-GSCs after 28 days of co-culture with CO/CCO. (D and E) GSCs were observed at the proximity of Iba1^+^ cells. (F and G) GSCs were in close contact to vascular-like CD31^+^ structures (enlarged views in F′ and G′). TG20 cells appeared to co-opt existing vascular structures. CCOs were derived from the PDF01 iPSC line (A, B, and D–G) and GM25256 iPSC line (C). Data are presented as median with interquartile range; each dot represents an individual CCO/CO. Comparisons across all groups were performed using the Kruskal-Wallis test with Dunn’s post hoc multiple-comparisons test; no significant differences were detected. CCOs and COs were compared pairwise using the Mann-Whitney test (#, *p* < 0.05).

GSCs were co-cultured with CCOs (45–60 DIV) for two days under orbital shaking to enrich for cells with active adhesion, after which the CCOs were separated from the non-adherent GSCs. Two weeks after co-culture, both TG16 and TG20 GSCs formed focal clones that were dispersed within the CCOs but were largely excluded from the densely packed neural rosettes (Figures 6A and 6B). To estimate the GSC percentage, organoids were dissociated after 28 days of co-culture and analyzed by FACS (Figure 6C). Despite the heterogeneity in GSC engraftment within each group, there was a trend toward higher EGFP-TG16 levels in CCOs compared with those in COs for both iPSC lines (*p* = 0.036 and *p* = 0.10 for GM25256 and PDF01, respectively; Mann-Whitney test), but no difference in GSC content in CCOs was observed between the iPSC lines. Resident microglial cells, which form part of the TAM population, along with vascular cells, play a critical role in GBM development and treatment resistance.^3^ Because iMG were widely present throughout the CCOs, they also located in close proximity to GSCs in the CCO co-cultures (Figures 6D and 6E). Additionally, both TG16 and TG20 GSCs were frequently observed in close association with ECs, with TG20 GSCs exhibiting signs of vessel co-option (Figure 6F).

Dissociation of the co-cultures enabled us to further quantify the effects of GSCs on ECs and iMG by FACS. The percentages of CD31^+^ ECs were not significantly altered by the presence of GSCs compared with CCO alone (Figure S9). However, in the TG16 GSC line, the expression of CD49d on CD31^+^ ECs was increased (Figure S9), consistent with observations in the GBM tumor core.^27^ The percentage of iMG was increased after co-culture with TG16 for both iPSC lines (Figure S9). Moreover, iMG displayed an immunosuppressive phenotype characterized by increased CD163 and CD206 expression in FACS analyses and upregulated MSR1 mRNA levels in sorted iMG (Figure S9). These phenotypic changes were further associated with the increased expression of SPP1, a TAM marker (Figure S9). Despite variability, iMG phenotypic alterations were observed in co-culture with GM25256-derived CCOs (Figure S9).

In summary, these results showed that co-cultures of GSCs with CCOs recapitulate several key features of the GBM tumor niche.

### The complexity of CCOs promoted the GBM-derived line aggressiveness after irradiation

**Figure 7:**
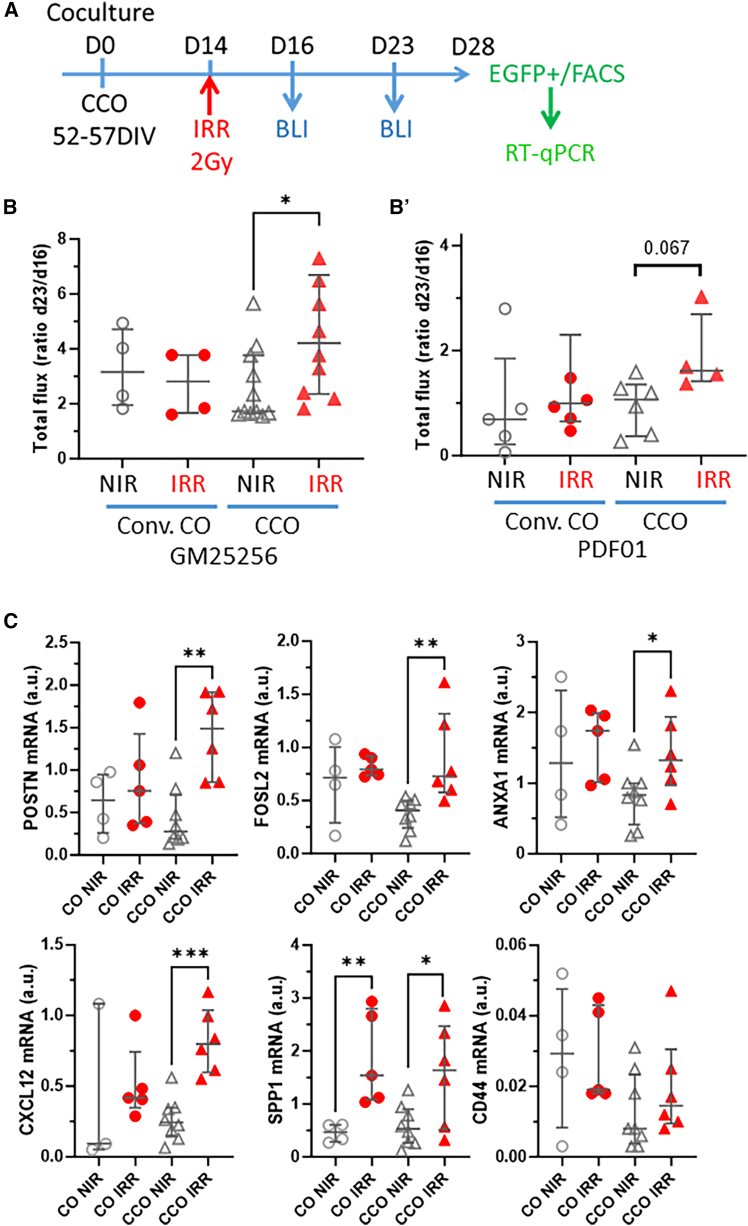
The complexity of the CCO niche promoted the TG16 GBM-derived line aggressiveness after irradiation (A and B) TG16-GSCs were co-cultured with CCOs for 14 days then were irradiated (IRR), or not (NIR). The amount of GSCs was determined by luciferase bioluminescence (photons/sec) 2 and 9 days later (D16 and D23). Then, the co-cultures were dissociated, and EGFP-GSCs were sorted by FACS. Longitudinal analyses of bioluminescence for GSCs co-cultured with conventional CO (Conv. CO) or complex CCO derived from the GM25256 iPSC line (B) and PDF01 iPSC line (B′). (C) EGFP-GSCs were sorted, and the expression of a set of NES genes was determined by RT-qPCR (a.u., arbitrary unit). CCOs were derived from PDF01 and GM25256 iPSC lines. Data are presented as median with interquartile range; each dot represents an individual CCO/CO. Comparisons across all groups were performed using the Kruskal-Wallis test with Dunn’s post hoc multiple-comparisons test (∗, *p* < 0.05; ∗∗, *p* < 0.01; ∗∗∗, *p* < 0.005).

Standard radiotherapy for GBM typically involves a total dose of 60 Gy, delivered in 2 Gy fractions. However, glioma cells at the margin of the irradiation field may receive lower doses. Additionally, invasiveness of glioma cells could be increased by irradiation,^28^ allowing them to escape the irradiation field. Thus, it is essential to consider the effects of lower doses, particularly the 2 Gy fractional dose, which may play a critical role in residual tumor progression. After two weeks of CCO-GSC co-culture, the co-cultures were exposed to a single 2 Gy dose of irradiation, and effects were analyzed two weeks later. Unirradiated TG16 cells exhibited a rounded morphology and formed clonal aggregates, whereas irradiated TG16 cells adopted a mesenchymal-like morphology and appeared more dispersed (Figure S10). We further quantified cell numbers by flow cytometry on dissociated co-cultures. Two weeks after 2 Gy exposure, total TG16 (GBM-derived GSC) counts showed a modest increase in GM25256-derived CCO co-cultures and a slight decrease in PDF01-derived CCO co-cultures (Figure S11). In contrast, TG20 GSCs exhibited a relative decrease after irradiation with CCOs from both iPSC lines (Figure S11D). However, relatively large variability in GSC engraftment, as reflected by EGFP cell recovery within each group, may have attenuated these effects. Therefore, to more robustly evaluate the effects of 2 Gy irradiation on GSC growth, we monitored bioluminescence of luciferase, encoded by the same vector as EGFP, in GSCs (Figure S11C). We measured the bioluminescence of TG16 and TG20 GSCs in CCOs on days 16 and 23 after co-culture, corresponding to days 2–9 post-irradiation, and compared it with those of conventional COs. The bioluminescence signal for TG16 and TG20 GSCs increased similarly in CCOs and conventional COs, indicating that GSCs developed comparably in each environment (TG16: Figure 7B and TG20: Figure S11D). However, irradiation favored TG16-GSC proliferation in CCOs, while it had no effect on TG16 in co-culture with conventional COs (Figures 7B and 7B′). In contrast, irradiation tended to reduce TG20 growth in either conventional COs or CCOs (Figure S11D).

We then sorted TG16 GSCs to analyze the expression of a set of genes associated with GBM development and aggressiveness, known as the natural evolution signature (NES).^29^ While CD44 mRNA levels were minimally impacted by irradiation, a specific set of genes (*SPP1*, *POSTN*, *FOSL2*, *ANXA1*, and *CXCL12*) were upregulated in TG16 cells co-cultured with CCOs following irradiation (Figure 7C). In contrast, with the exception of *SPP1*, the expression of these genes remained largely unchanged in TG16 tumors derived from conventional COs (Figure 7C). This gene alteration aligned with the observed radiation-induced proliferation of TG16 GSCs and their mesenchymal phenotype.

We then analyzed the effects of irradiation on iMG/TAMs, using FACS. While the proportion of iMG remained unchanged in CCOs (Figure S12), the expression of HLA-DR, CD11b, CD163, and CD206 on iMG increased following irradiation, indicating their alternative activation toward an immunosuppressive TAM phenotype (Figure S12). This radiation-induced response was consistently observed in co-cultures with GBM-derived GSCs (TG16) across both iPSC lines, while co-cultures with astrocytoma-derived GSCs (TG20) exhibited only a limited effect (Figure S12).

## Discussion

To generate CCOs that contained both vascular structures and microglia-like cells, we replicated the stages of embryoid brain development, focusing on the colonization of COs by ECs and primitive macrophages. We, thus, incorporated bipotent hemato/ECs during the early stages of CO formation. We validated these vascularized, immunocompetent COs to model the GBM tumor niches and assess resistance to radiotherapy.

Given the critical role of vascularization in tissue physiopathology, recent efforts have focused on vascularizing COs. Brain vasculature is formed by a combination of ECs, astrocytes, pericytes, and neurons, creating the BBB, an impermeable structure.^30^ Most BBB cell types, such as astrocytes, pericytes, and neurons, are derived from the neuroepithelium and can arise spontaneously within COs. However, conventional COs lack the ability to generate vascular ECs, as these cells originate from the mesoderm.

In this study, we added iPSC-derived HECs along with exogenous VEGF and BMP4 to initiate EC differentiation. We anticipated that neural progenitor cells within the organoids would produce signals necessary for inducing vascular network formation.^31^ Indeed, we observed vascular-like structures, identified by the EC marker CD31, surrounded by basal lamina (collagen IV) and pericyte coverage (PDGFRβ). Additionally, the presence of ZO-1, a key component of the tight junctions associated with BBB, was confirmed.

Recent studies have also modeled vascularized brain organoids with BBB features by fusing vascular organoids and COs *in vitro.*^19^^,^^32^ However, these vascular structures initially lacked a lumen, leading to apoptosis in the absence of blood flow. Consistent with previous findings,^14^ once our CCOs were transplanted into immunodeficient mice, the vascular structures became perfused with blood, confirming their functionality. We believe that the presence of vascular structures will further enable the integration of CCOs into microfluidic chips for advanced research applications.^33^ In a previous study, iPSCs expressing ETV2 contributed to the formation of a complex vascular network within CCOs and enhanced the functional maturation of the organoids.^14^ In our model, the inclusion of HECs enabled the generation of a substantial number of neurons within the CCOs. Neural rosettes, i.e., neural progenitor-rich regions, were less abundant in our CCOs compared with previously reported conventional COs, likely reflecting a more advanced maturation state associated with the integration of HECs. Moreover, we observed significant numbers of astrocytes (S100β/GFAP) as early as days 60–80, but astrocyte abundance is typically reported to increase after 90–100 days.^34^^,^^35^ This suggests that the combination of vascularization and microglia supports the maturation of COs.

Microglial cells play a critical role in regulating neuronal wiring, angiogenesis, and immune responses in the brain, originating from extraembryonic erythroid-myeloid progenitors.^36^ Various techniques have been used to generate COs containing microglial cells.^16^ In this study, by incorporating HECs into COs derived from the same iPSC lines, we successfully produced COs with iMG. Given their expression of CD309 and CD144, iPSC-derived HECs are closely related to erythroid-myeloid progenitors, from which microglia originate.^23^ Although CCOs appeared to intrinsically produce IL-34 and CX3CL1/Fractalkine, both of which are involved in microglia homeostasis, the organoid medium was supplemented with GM-CSF and IL-34 to support the long-term maintenance of iMG.

The brain environment significantly influences the expression profile of microglial cells, distinguishing them from macrophages.^37^ In our CCO model, iMG cells expressed key surface markers such as CX3CR1, P2RY12, and TMEM119 and displayed specific gene expression patterns consistent with previous studies.^17^^,^^18^ Notably, in our FACS experiments, iMG cells represented 0.50% (0.16–1.42) of the total cell population within CCOs, aligning with the levels of microglia observed in the adult human brain, which range from 0.5% to 16.6%, depending on the brain region.^38^ The iMG cells in our CCOs exhibited a ramified morphology that transitioned from a rudimentary, initial state, to a more complex structure over the culture period. We demonstrated that these microglia-like cells exhibited characteristics of natural microglia, including the ability to engulf neuronal synapses (PSD95 expression), in agreement with previous findings.^16^^,^^17^^,^^19^

Recently, Sun et al. reported a model integrating both ECs and microglia simultaneously.^19^ In contrast to their approach, which involved generating vascular organoids, we adopted a different strategy by dissociating HEC colonies. Overall, our results corroborate their findings regarding the integration of ECs and microglia in CCOs. Nevertheless, using dissociated HECs instead of vascular organoids may offer specific technical advantages, such as facilitating the manipulation of specific cell populations (treatment, electroporation).

After validating our CCO model, we used it to explore the tumor microenvironment (TME) of GBM by co-culturing CCOs with GSC lines. The tumor microenvironment includes various classes of non-neoplastic tissue-resident and infiltrating cells.^3^ The non-neoplastic brain environment promotes electrochemical coupling between tumors and neurons, as well as metabolic interactions with astrocytes, which drive tumor growth.^3^ Neurogenic niches have been proposed as a favorable environment for glioma cells,^39^ although the proximity of glioma cells to these niches may also reflect their stem cell origin.^40^ In our observations, we did not find any preferential location or development of GSCs near the neurogenic niches of CCOs, particularly neural rosettes. Instead, we observed that in co-cultures with CCOs, GSCs were frequently surrounded by numerous iMG cells (Iba1^+^).

TAMs represent the largest population of non-cancerous cells in the GBM tumor microenvironment, comprising up to 30%–50% of the tumor mass.^5^ This population includes a mix of resident microglia and macrophages derived from infiltrating monocytes.^7^ While the infiltration of blood-derived TAMs can be inhibited using CSF1R inhibitors or chemokine receptor antagonists,^41^ resident microglia persist in the brain and can acquire TAM-like features. GBM cells can hijack microglial gene expression to support tumor growth,^42^ causing TAMs to adopt immunosuppressive properties.^43^ Interactions between cancer cells and immune cells are known to drive transition to mesenchymal-like states in GBM.^44^

Our model allows for the specific investigation of how GSCs influence microglial cells. Indeed, we demonstrated that co-culturing CCOs with GSCs alters the phenotype of iMG, particularly inducing them to adopt an immunosuppressive state, as evidenced by the increased expression of markers such as CD163, CD206, MSR1, and the TAM-associated gene *SPP1*. The higher expression levels of factors that influence TAMs in the TG16 line, such as CSF1,^41^ CCL2, and POSTN,^45^ likely explain the more pronounced alterations in iMG in the presence of TG16 compared to TG20.

Additionally, GBM cells were often found in close proximity to vascular structures, suggesting vessel co-option in TG20 cells.^46^ Moreover, ECs in the co-cultures showed elevated CD49d expression, a feature that has been previously reported in the tumor core.^27^ These effects of GSCs on iMG and EC phenotypes showed variability, likely reflecting the fact that some iMG or ECs were not in close proximity to glioma cells, as the latter were not widely distributed throughout the CCOs. Therefore, further studies, such as those employing spatial transcriptomic, are warranted.

Radiotherapy is administered to nearly all GBM patients, yet it may enhance the invasiveness of GSCs,^28^ which are highly resistant to radiation.^1^ GSCs can infiltrate brain parenchyma and persist after surgical resection and radiotherapy.^47^ Those located at the tumor margin are often exposed to lower doses of radiation compared with the central tumor mass. We demonstrated that GSCs proliferate in co-cultures with conventional COs, and radiation exposure does not hinder their growth, consistent with previous studies using even higher doses of radiation.^13^ Remarkably, we observed that irradiation of co-cultures with CCOs actually promoted GSC proliferation. Although we cannot definitively pinpoint the specific cell population(s) responsible for this radiation-induced effect on GSCs, the enhanced recovery of GSCs was evident only in immunocompetent vascularized CCOs, not in conventional COs, suggesting a possible role for TAMs and/or vascular cells, an assumption that requires further confirmation. This observation is reminiscent of the recurrences seen within 1–2 cm of the primary tumor border in GBM patients.^48^ Similarly, in experimental GBM models of mice, initial depletion of tumor cells post-irradiation was often followed by significant recurrence, which is correlated with tumor microenvironment reorganization, particularly involving TAMs.^6^^,^^49^ Interactions between cancer and immune cells drive transition to mesenchymal-like states in GBM.^44^

Radiation also induces persistent reprogramming of microglia into a primed state^50^ and expands TAM populations with an immunosuppressive profile.^51^ Notably, we observed that iMG were already reprogrammed toward an immunosuppressive TAM state in the presence of TG16 GSCs. A gene expression signature, known as NES, has been identified in patients and is significantly associated with tumor evolution and macrophage polarization.^29^ Intriguingly, several NES genes were upregulated after irradiation in TG16 GSCs co-cultured with CCOs. Among these, *POSTN* plays a dual role in maintaining GBM stem cells and promoting the immunosuppressive phenotype of microglia in GBM.^45^ The immunosuppressive environment created by TAMs is responsible for the failure of immunotherapies.^52^ Therefore, our model will be invaluable for elucidating the mechanisms driving recurrence after radiotherapy and for discovering therapeutic strategies, particularly in the context of precision medicine.

Primary brain tumors, particularly pediatric and adult diffuse gliomas, are strongly influenced by the tumor microenvironment, including microglia, TAMs, and vascular cells. Recent studies have introduced patient-derived tumoroids, or GBM organoids, generated from tumor explants, which recapitulate key histological features, cellular heterogeneity, and mutational profiles of the parental tumors and enable biobanking for personalized therapeutic testing.^53^^,^^54^ Despite the partial loss of patient-derived immune and vascular components during culture, tumoroids remain valuable preclinical models to predict disease progression and therapeutic response.^54^^,^^55^^,^^56^^,^^57^ More recently, CO-based models have enabled the investigation of tumor invasion and cellular plasticity within a human neural context.^13^ Co-culture of human GSCs with COs has been valuable to study tumor growth and identify invasion signatures that closely resemble those observed in surgical GBM specimens.^13^^,^^58^ This model also provided insight into the intercellular communications between malignant and non-malignant cells.^59^ However, such models present important limitations, notably the absence of vascular and immune compartments. Only recently have more complex CO systems begun to incorporate vasculature and/or microglia to address these shortcomings, although these approaches remain technically demanding and have not been widely adopted yet. Very recently, neuroimmune-competent human brain organoid glioma models have been reported using either microglia-containing brain organoids^60^ or the integration of matched peripheral blood mononuclear cells into human cortical organoids, enabling the study of patient-specific immune responses and tumor-immune interactions.^61^ Within this landscape, our approach simultaneously integrates glioma cells with both microglial and endothelial components in a single, fully human 3D system that more closely reflects the *in vivo* situation. Our model supports long-term culture, enabling in-depth analyses of tumor development and progression and the effects of diverse treatments and the incorporation of multiple cell types, genetically modified or not. It may also reproduce invasion of glioma cells into normal brain tissue. To our knowledge, no other iPSC-derived CO incorporating both microglia and vascular cells has been used to study gliomas.

In conclusion, our vascularized and immunocompetent CCO model offers a robust platform for investigating cellular interactions within the tumor microenvironment and assessing therapeutic responses. Additionally, it holds significant potential for advancing our understanding of human cerebral development and the disruptions that occur in diseases involving the endothelium and microglia.

### Limitations of the study

The developmental state of COs has been reported to approximate late embryonic stages. In our model of CCOs, their cellular complexity favored astrocyte production, which may impart features reminiscent of a more advanced developmental stage. Further works are, therefore, warranted to precisely define the developmental stage of our CCOs.

*In vitro*, the vascular structures in CCOs lack lumen, but blood flow is known to promote vessel maturation. Notably, following transplantation into mice, these vessels exhibited blood flow and pericyte coverage, indicating more advanced maturation *in vivo*. These transplantation experiments were performed exclusively in male mice and should be extended to include female mice. Such transplantation strategies provide a useful avenue to study human-specific neuro-immune interactions under *in vivo* conditions. In parallel, engineering more sophisticated human organoid models within microfluidic devices may enable investigations under conditions that more closely mimic the *in vivo* environment.

In this study, cell populations in CCOs were quantified by FACS analysis of the dissociated whole organoids, while their characterization was performed by immunohistology on thick 3D sections. Although technically challenging, the quantitative analysis of these cell populations by immunohistology on intact organoids—which preserves spatial organization—warrants further development.

The direct involvement of ECs and microglia in the development of tumoral cells has not been demonstrated in this work and will be further assessed in future works.

Finally, the number of GSCs maintained in co-culture with CCOs is substantially lower than that in other model such as tumor explants. This suggests that our model more closely reflects the invasive behavior of tumor cells within normal brain tissue, rather than the cellular composition of the tumor core.

## Resource availability

### Lead contact

Requests for further information should be directed to and will be fulfilled by the lead contact, Marc-André Mouthon (marc-andre.mouthon@cea.fr).

### Materials availability

This study did not generate new unique reagents.

### Data and code availability

• All data reported in this paper will be shared by the lead contact upon request.
• This study does not report original code.
• Any additional information required to reanalyze the data reported in this paper is available from the lead contact upon request.

## Acknowledgments

We are indebted to M.T. Garcia-Mitjavila, L. Morizur, and A. Perrier for iPSC lines and advice on iPSC cultures and organoid generation; C. Duwatt, V. Barroca, and S. Devanand for mice experiments; S. Messiaen for multiplex experiments; F. Pflumio for giving immunodeficient mice; and V. Ménard for irradiation. J.R. and O.B. are fellows from CEA (CFR « blanc» and CFR Organoid on Chip). J.R. is an ImmunoTools Award winner. This work was supported by 10.13039/501100004099La Ligue Contre le Cancer (comité des Hauts-de Seine), Les Entreprises Contre le Cancer (GEFLUC), 10.13039/501100016036Electricité de France, and GIS FC3R (project no. 22FC3R-024) from funds managed by 10.13039/501100001677INSERM. IStem/CECS is supported by 10.13039/100007393Association Française contre les Myopathies (AFM-Téléthon).

## Author contributions

M.-A.M. conceptualized the study; L.C., T.K., and O.B. helped in methodology; C.G.-B., J.R., N.L., and M.-A.M. did experiments and formal analyses; L.C. and A.B. supplied biological resources; J.R., N.L., and M.-A.M. prepared figures and wrote the original draft; L.R.G., F.D.B., and M.-A.M. reviewed and edited the manuscript; L.R.G., F.D.B., M.-A.M., and A.B. obtained funding; and F.D.B. and M.-A.M. supervised the study. All authors reviewed the manuscript.

## Declaration of interests

The authors declare no competing interests.

## STAR★Methods

### Key resources table

**Table:** 

REAGENT or RESOURCE	SOURCE	IDENTIFIER
**Antibodies**		

CD11b-BB700	BD Biosciences	Clone identity: M1/70 Cat#566417 RRID: AB_2744272
CD11b-BV421	BioLegend	Clone identity: M1/70 Cat#101235 RRID: AB_10897942
CD31-BV605	BioLegend	Clone identity: WM59 Cat#303122 RRID: AB_2562148
CD31-PE	Immunotools	Clone identity: MEM-05 Cat#21270314S RRID: AB_3662732
CD43-PE-Cy7	BioLegend	Clone identity: CD43-10G7 Cat#343208 RRID: AB_2563698
CD45-APC	BioLegend	Clone identity: HI30 Cat#304012 RRID: AB_2562049
CD45-PE	BioLegend	Clone identity: HI30 Cat#304008 RRID: AB_2562057
CD49d-APC-Cy7	BioLegend	Clone identity: 9F10 Cat#304327 RRID: AB_2616848
CD144-APC	BioLegend	Clone identity: BV9 Cat#348507 RRID: AB_10639861
CD146-BV605	BioLegend	Clone identity: P1H12 Cat#361024 RRID: AB_2783271
CD163-BV510	BioLegend	Clone identity: GHI/61 Cat#333628 RRID: AB_2650631
CD206-APC	Thermo Fisher Scientific	Clone identity: 19.2 Cat#17-2069-42 RRID: AB_2573182
CD309-BV421	BioLegend	Clone identity: A16085H Cat#393010 RRID: AB_2832739
CX3CR1-APC	Thermo Fisher Scientific	Clone identity: 2A9-1 Cat#17-6099-42 RRID: AB_11149136
HLADR-SB780	Thermo Fisher Scientific	Clone identity: LN3 Cat#78-9956-42 RRID: AB_2724457
P2RY12-BV421	BioLegend	Clone identity: S16001E Cat#392105 RRID: AB_2783290
SSEA3-AF488	R and D Systems	Clone identity: MC-631 Cat#FAB1434G RRID: AB_3646484
TRA-1-81-AF594	R and D Systems	Clone identity: TRA-1-81 Cat#IC8495T RRID: AB_3662726
AIF-1/Iba1	Novus	Clone identity: polyclonal Cat#NBP2-19019 RRID: AB_3073939
AIF-1/Iba1	Novus	Clone identity: polyclonal Cat#NB100-1028 RRID: AB_521594
CD31	Biolegend	Clone identity: P2B1 Cat#910005 RRID: AB_2566676
Collagen IV	Abcam	Clone identity: polyclonal Cat#ab6586 RRID: AB_305584
Connexin 43	Abcam	Clone identity: polyclonal Cat#ab11370 RRID: AB_297976
Doublecortin (DCX)	Novus	Clone identity: polyclonal Cat#NBP1-72042 RRID: AB_11019667
GFAP	Cell Signaling Technology	Clone identity: GA5 Cat#3670 RRID: AB_561049
GFP	Novus	Clone identity: polyclonal Cat#NB100-1614 RRID: AB_10001164
GFP	Novus	Clone identity: polyclonal Cat#NB100-1770 RRID: AB_10128178
MAP2	Millipore	Clone identity: AP20 Cat#MAB3418 RRID: AB_94856
P2RY12	Sigma-Aldrich	Clone identity: polyclonal Cat#HPA014518 RRID: AB_2669027
PDGFRβ	R and D Systems	Clone identity: polyclonal Cat#AF1042 RRID: AB_2162633
PSD95	Cell Signaling Technology	Clone identity: polyclonal Cat#3450 RRID: AB_2292883
S100β	Agilent DAKO	Clone identity: polyclonal Cat#Z0311 RRID: AB_10013383
SOX2	Millipore	Clone identity: polyclonal Cat#AB5603 RRID: AB_2286686
SOX9	Millipore	Clone identity: polyclonal Cat#AB5535 RRID: AB_2239761
ZO-1	Thermo Fisher Scientific	Clone identity: ZO1-1A12 Cat#33–9100 RRID: AB_2533147
β3-tubulin	BioLegend	Clone identity: TUJ1 Cat#801202 RRID: AB_2313773
Rabbit anti-Chicken-FITC	Thermo Fisher Scientific	Clone identity: polyclonal Cat#SA1-9511 RRID: AB_1075130
Donkey anti-Goat-AFplus488	Thermo Fisher Scientific	Clone identity: polyclonal Cat#A32814 RRID: AB_2762838
Donkey anti-Goat-AF594	Thermo Fisher Scientific	Clone identity: polyclonal Cat#A-21468 RRID: AB_2535871
Donkey anti-Goat-AF647	Thermo Fisher Scientific	Clone identity: polyclonal Cat#A-21447 RRID: AB_141844
Chicken anti-Mouse-AF488	Thermo Fisher Scientific	Clone identity: polyclonal Cat#A-21200 RRID: AB_2535786
Donkey anti-Mouse-AF488	Thermo Fisher Scientific	Clone identity: polyclonal Cat#A-21202 RRID: AB_141607
Donkey anti-Mouse-AF594	Thermo Fisher Scientific	Clone identity: polyclonal Cat#A-21203 RRID: AB_2535789
Donkey anti-Mouse-AFplus647	Thermo Fisher Scientific	Clone identity: polyclonal Cat#A32787 RRID: AB_2762830
Donkey anti-Rabbit-AF488	Thermo Fisher Scientific	Clone identity: polyclonal Cat#A-21206 RRID: AB_2535792
Donkey anti-Rabbit-AF594	Thermo Fisher Scientific	Clone identity: polyclonal Cat#A21207 RRID: AB_141637
Donkey anti-Rabbit-AFplus647	Thermo Fisher Scientific	Clone identity: polyclonal Cat#A32795 RRID: AB_2762835
OmniMap anti-Ms HRP	Roche	Cat#5269652001; RRID: AB_2885182
OmniMap anti-Rb HRP	Roche	Cat#5269679001
OPAL520	AKOYA Biosciences	Cat#FP1487001KT
OPAL570	AKOYA Biosciences	Cat#FP1488001KT
OPAL620	AKOYA Biosciences	Cat#FP1495001KT
CD31	Biolegend	Clone identity: WM59 Cat#303102 RRID: AB_314328
GFAP	Millipore	Clone identity: GA5 Cat#IF03L RRID: AB_2294571
LAMININ a4	Millipore	Clone identity: 4B12 Cat#MABT39 RRID: AB_10807830

**Chemicals, peptides, and recombinant proteins**		

StemMACS™ iPS-Brew XF, human	Miltenyi Biotec	Cat#130-104-368
Vitronectin	Gibco™	Cat#A14700
mTESR1	Stem Cell Technologies	Cat#85850
Corning® Matrigel® hESC-Qualified Matrix, LDEV-free	Corning	Cat#354277
EDTA	Invitrogen	Cat#15575-038
Accutase™	Sigma Aldrich	Cat# A6964
STEMdiff™ APEL™2	Stem Cell Technologies	Cat# 05275
Y-27632	Stem Cell Technologies	Cat#7230
Human VEGF-165 Recombinant Protein	PeproTech®	Cat#100-20-050u
Human BMP-4 Recombinant Protein	PeproTech®	Cat#120-05-100u
CHIR99021	BioTechne	Cat#4423
Human Flt-3 Ligand (FLT3L) Recombinant Protein	PeproTech®	Cat#300-19-10u
Human SCF Recombinant Protein	PeproTech®	Cat#300-07-10u
Human TPO (Thrombopoietin) Recombinant Protein	PeproTech®	Cat#300-18-10u
TrypLE™ Express	Gibco	Cat#12605010
DMEM/F12 1% Glutamax	Gibco	Cat#31331028
Knockout Serum Replacer	Gibco	Cat#10828028
MEM-NEAA	Gibco	Cat#11140050
2β-mercaptoethanol	Gibco	Cat# 31350010
Fetal Bovine Serum	Gibco	Cat#10500064
Anti-Anti	Gibco	Cat#15240062
thermostable human basic fibroblast growth factor (bFGF)	Gibco	Cat#PHG0360
N2	Gibco	Cat#17502048
Heparin	Stem Cell Technologies	Cat#07980
Corning® Matrigel® Growth Factor Reduced (GFR) Basement Membrane Matrix, LDEV-free	Corning	Cat#354230
Neurobasal	Gibco	Cat#21103049
Glutamax	Gibco	Cat#35050061
Insulin	Sigma-Aldrich	I9278
B27 without vitamin A	Gibco	Cat#12587010
Human IL-34 Recombinant Protein	PeproTech®	Cat#200-34
Human GM-CSF Recombinant Protein	PeproTech®	Cat#300-03
B27	Gibco	Cat#17504044
Papain	Worthington	Cat#LS003119
DNAse I	Sigma Aldrich	Cat#D5025
EDTA	Sigma Aldrich	Cat#E5134
L-cysteine	Sigma Aldrich	Cat#C7352
EBSS	Gibco	Cat#24010043
Trypsin inhibitor type II	Sigma Aldrich	Cat#T9128
bisBenzimide H 33258	Sigma Aldrich	Cat#23491-45-4 H
human recombinant epidermal growth factor (EGF)	Sigma Aldrich	Cat#E9644
D-luciferin	PerkinElmer	Cat#122799
70kDa FITC-dextran	Sigma Aldrich	Cat#90718
RLT lysis buffer	Qiagen	Cat#79216
RNeasy Micro Kit with DNase treatment	Qiagen	Cat#74004
NucleoSpin RNA XS	Marcherey-Nagel	Cat#740902
Reverse Transcription High Capacity Master Mix	Applied Biosystems	Cat#4368814
Power SYBR GREEN	Applied Biosystems	Cat#4367659
BSA	Sigma Aldrich	Cat#A7906
Fluoromount-DAPI	Southern Biotechnologies	Cat#0100-20
PBS-TABS	Sigma Aldrich	Cat#18912
Triton X-100	Sigma Aldrich	Cat#T8787
BD Perm/Wash Buffer	BD Bioscience	Cat#554723
Donkey Serum	Eurobio Scientific	Cat#CAEMTN00-0U
1-thioglycerol	Sigma Aldrich	Cat#M1753
Saponine	Sigma Aldrich	Cat#S-4521
DAPI	Sigma Aldrich	Cat#D9542

**Experimental models: Cell lines**		

PDF01	ATCC®, ISTEM, Evry	PCS-201-012™
GM25256∗G	Coriell	Cat#GM25256, RRID:CVCL_Y803

**Experimental models: Organisms/strains**		

Male NOD-SCID mouse: NOD.Cg-*Prkdc*^*scid*^*Il2rg*^*tm1Wjl*^/SzJ	The Jackson Laboratory	RRID:IMSR_JAX:005557
Nude mouse: Rj:NMRI-Foxn1 nu/nu	Janvier Labs	SM-NMRNU-F

**Oligonucleotides**		

qRT-PCR primers used in this work	This paper	see Table S1

**Software and algorithms**		

FlowJo	BD Bioscience	RRID:SCR_008520
StepOne	Applied Biosystems	RRID:SCR_014281
Leica Application Suite X	Leica	RRID:SCR_013673
IMARIS	Bitplane	RRID:SCR_007370
GraphPad Prism	GraphPad	RRID:SCR_002798

**Other**		

24-wells AggreWell™800	Stem Cell Technologies	Cat#34815
Corning® 96-well Clear Round Bottom Ultra-Low Attachment Microplate	Corning	Cat#7007
24-well plate	Falcon	Cat#353047
Bioluminescence Imager (IVIS LUMINA II)	Perkin Elmer	IVIS LUMINA II
BD FACSAria II Cell Sorter	BD Bioscience	RRID:SCR_018934
GSR D1 irradiator	Gamma services	GSR D1
Applied Biosystems StepOne Real-Time PCR System	Applied Biosystems	RRID:SCR_023455
Tissue-Tek	Sakura	Tissue-Tek
microtome	Leica Microsystems	RM2125RT
SuperFrost Plus slides	Epredia	Cat#J1800AMNZ
Roche Ventana BenchMark ULTRA IHC/ISH System	Roche	RRID:SCR_025506
Leica SP8 LIGHTNING confocal microscope	Leica	RRID:SCR_018169
Spinning-Disk confocal microscope	Nikon, GATTACA	N/A
1mm piezoelectric	Piezoconcept	N/A
Leica VT1200S vibrating microtome	Leica	RRID:SCR_020243
24 well plate glass bottom	Azenta	Cat#4TI-0243
cover slips	Epredia	Cat#CB00120RA120MNZ0

### Experimental model and study participant details

#### Animals

All animal experiments described in this study were approved by the Institutional Animal Care & Use Committee of CEA/DRF (CEtEA) and were performed under the EU guidelines with approved protocol (APAFIS#40886–2023021010503692). NOD-SCID mice (RRID:IMSR_JAX:005557) were bred in our animal facility. Three-to five-month-old male from different littermates of the same sex were randomly assigned to experimental groups.

#### Cell lines

Human iPSC lines PDF01 (reprogrammed from dermal female fibroblasts (ATCC #PCS-201-012) at ISTEM, Evry, France) and GM25256∗G (derived from male fibroblasts; Coriell Cat#GM25256, RRID:CVCL_Y803) were cultured, respectively, in IPS-BREW XF (Miltenyi Biotec Cat#130-104-368, StemMACS iPS-Brew XF, human) on Vitronectin (GibcoTM Cat#A14700, VTN-N) coated dishes and in mTESR1 (Stem Cell Technologies Cat#85850, mTeSR1) on Matrigel (Corning Cat#354277, Corning Matrigel hESC-Qualified Matrix, LDEV-free) coated dishes. iPSCs were passaged and used at 70–80% of confluence. Pluripotency was routinely checked for >85% TRA1-81 (R and D Systems Cat# IC8495T, RRID:AB_3662726) and SSEA3 (R and D Systems Cat#FAB1434G, RRID:AB_3646484) staining by flow cytometry.

The TG16 and TG20 human GSC lines were established from surgical resections carried out at Sainte Anne Hospital (Paris, France)^26^^,^^62^ on males with high-grade IDH wt/mut gliomas according to the WHO classification (GBM: TG16; astrocytoma: TG20). TG20 line is mutated for TP53 (P278L), loss of ATRX but no deletion of 1p/19q. GSC lines were cultured as gliospheres in defined stem cell culture condition (serum-free Dulbecco’s Modified Eagle Medium DMEM/F12 supplemented with 1% Anti-Anti, 1x B27 without vitamin A, 5 μg/mL heparin, 20 ng/mL human recombinant epidermal growth factor (EGF, Sigma Aldrich Cat#E9644) and 20 ng/mL bFGF at 37°C in an atmosphere containing 5% CO2. Every week, cells were dissociated after a 5 min incubation at room temperature with the Accutase cell dissociation reagent and reseeded at 0.5 × 10^6^ cells per T75 flask. GSC lines were transduced using a lentiviral vector containing a Luciferase/EGFP cassette (pTRIP-MND-Luciferase-Ires-GFP).^62^

### Method details

#### Hemogenic endothelial cell differentiation

Hemogenic endothelial cells (HECs) were generated according to Vargas-Valderamma’s protocol with some modifications.^23^ Briefly, iPSC colonies were moderately dissociated with 0.5mM EDTA (Invitrogen Cat#15575-038) and, optionally with AccutaseTM (Sigma Aldrich, Cat# A6964). One million of cells were cultured in 5mL STEMdiff APEL2 medium (Stem Cell Technologies, Cat# 05275) in the presence of 10μM Y-27632 (Stem Cell Technologies Cat#7230), 50ng/mL Human VEGF-165 Recombinant Protein (PeproTech Cat#100-20-050u) 50ng/mL Human BMP-4 Recombinant Protein (PeproTech Cat#120-05-100u), 6μM CHIR99021 (BioTechne Cat#4423) in a 25 cm^2^ flask. Alternatively, GM25256 cells for which dissociation is critical, HEC cultures were cultured into 24-wells AggreWell800 (Stem Cell Technologies Cat#34815) with 500 cells/μwell. After 48h, cells were transferred to APEL2 medium with 50ng/mL VEGF-165, 50ng/mL BMP-4, 20ng/mL Human Flt-3 Ligand (FLT3L) Recombinant Protein (PeproTech Cat#300-19-10u), 20ng/mL Human SCF Recombinant Protein (PeproTech Cat#300-07-10u) and 20ng/mL Human TPO (Thrombopoietin) Recombinant Protein (PeproTech Cat#300-18-10u) for 3 or 4 days. Five to seven days after their initiation, HECs were recovered from adherent clones by dissociation with TrypLE Express (Gibco Cat#12605010) or Accutase.

#### Obtaining of complex cerebral organoids

Human cerebral organoids were generated according to Lancaster’s protocol with modifications.^25^ Briefly, iPSC colonies were dissociated with 0.5mM EDTA and Accutase. Embryoid bodies (EB) were formed with 9,000 cells in Corning 96-well Clear Round Bottom Ultra-Low Attachment Microplate (Corning Cat#7007) and cultured in hESC medium (DMEM/F12 1% Glutamax [Gibco Cat#31331028] containing 20% [v/v] Knockout Serum Replacer [Gibco Cat#10828028], 1% [v/v] MEM-NEAA [Gibco Cat#11140050], 100μM 2β-mercaptoethanol [Gibco Cat# 31350010], 15% Fetal Bovine Serum [Gibco Cat#10500064], 1% [v/v] Anti-Anti [Gibco Cat#15240062]) with 10 μM Y-27632 and 4ng/mL thermostable human basic fibroblast growth factor (bFGF, Gibco Cat#PHG0360). The fifth day, EB were transferred in hESC medium without bFGF or Y-27632 for 6 h. Then, EB were transferred in Neural Induction Medium (DMEM/F12 1% Glutamax containing 1% [v/v] N2 [Gibco Cat#17502048], 1% [v/v] MEM-NEAA, 1μg/mL Heparin [Stem Cell Technologies Cat#07980], 1% [v/v] Anti-Anti). Seven days after their initiation, embryoid bodies were cocultured for 2 days with dissociated HEC (10^4^ HEC/embryoid body) in neural induction medium under static conditions. Then, complex cerebral embryoid bodies were embedded into 20μL droplet of GFR Matrigel (Corning Cat#354230) and cultivated into cerebral organoid differentiation medium (50% [v/v] DMEM/F12 with 50% [v/v] Neurobasal [Gibco Cat#21103049] containing 1% [v/v] Glutamax [Gibco Cat#35050061], 0.5% [v/v] N2, 2.63 μg/mL Insulin [Gibco Cat#], 0.5% [v/v] MEM-NEAA, 50μM 2β-mercaptoethanol) composed of 0.5% (v/v) B27 without vitamin A (Gibco Cat#12587010) supplemented with 20ng/mL VEGFA, 100ng/mL IL-34 (PeproTech Cat#200-34), 10ng/mL GM-CSF (PeproTech Cat#300-03). After 2–3 days, the medium was replaced by cerebral organoid differentiation medium with 0.5% (v/v) B27 (Gibco Cat#17504044), 20ng/mL VEGFA, 100ng/mL IL-34, 10ng/mL GM-CSF and renewed each 3–4 days. CCOs were kept under orbital shaking from day 11 (85 rpm).

#### Obtaining of cerebral organoids

Conventional cerebral organoids were generated following the same protocol as CCOs, but without the addition of human endothelial cells (HECs) or growth factors (VEGF, IL-34, and GM-CSF).

#### Subcutaneous implantation of organoids into mice

Thirty days after initiation, CCOs were implanted into the leg of NOD-SCID mice. Mice were placed into a chamber by providing 2% isoflurane for anesthetization. Then, mice were immobilized under a laminar flow hood to cut a small incision at each back leg. CCOs were subcutaneously placed into the incision of the right and left back legs. Once CCO were inserted, the wound was closed with sutures and buprenorphine (0.1 mg/kg) was administered for pain relief. One month after xenotransplantation, mice were anesthetized and perfused with 25 mL of 70kDa FITC-dextran (Sigma Aldrich Cat#90718) for 5 min then euthanized. Subsequently, CCOs were recovered and fixed in 4% PFA for immunostaining.

#### Flow cytometry

CCOs were dissociated into single cell for FACS analyses. Briefly, CCOs were minced into small pieces by razor blade and incubated 30 min at 37°C, under orbital shaking (85 rpm), with 30U/mL Papain (Worthington Cat#LS003119), 100U/mL DNAse I (Sigma Aldrich Cat#D5025), 0.05mM EDTA (Sigma Aldrich Cat#E5134) and 0.18mg/mL L-cysteine (Sigma Aldrich Cat#C7352) in EBSS (Gibco Cat#24010043). After mechanical dissociation, cell suspensions were further incubated for 5–10 min at 37°C under orbital shaking. Then, cells were filtered into single cell in 10mg/mL Trypsin inhibitor type II (Sigma Aldrich Cat#T9128) and 100U/mL DNAse I (Sigma Cat#D5025).

Dissociated cells were incubated for 15 min at 4°C with fluorochrome-labeled antibodies in PBS containing 0.15% BSA. Then, cells were washed and resuspended in 0.15% BSA/PBS with 1μg/mL of bisBenzimide H 33258 (Sigma Aldrich Cat#23491-45-4 H) and sorted on a BD FACSAria II Cell Sorter (RRID:SCR_018934). Data were analyzed on FlowJo (RRID:SCR_008520).

#### Glioma stem cell cultures

The TG16 and TG20 GSC lines were established from surgical resections carried out at Sainte Anne Hospital (Paris, France)^26^^,^^62^ on patients with high-grade IDH wt/mut gliomas according to the WHO classification (GBM: TG16; astrocytoma: TG20). TG20 line is mutated for TP53 (P278L), loss of ATRX but no deletion of 1p/19q. GSC lines were cultured as gliospheres in defined stem cell culture condition (serum-free Dulbecco’s Modified Eagle Medium DMEM/F12 supplemented with 1% Anti-Anti, 1x B27 without vitamin A, 5 μg/mL heparin, 20 ng/mL human recombinant epidermal growth factor (EGF, Sigma Aldrich Cat#E9644) and 20 ng/mL bFGF at 37°C in an atmosphere containing 5% CO2. Every week, cells were dissociated after a 5 min incubation at room temperature with the Accutase cell dissociation reagent and reseeded at 0.5 × 106 cells per T75 flask. GSC lines were transduced using a lentiviral vector containing a Luciferase/EGFP cassette (pTRIP-MND-Luciferase-Ires-GFP).^62^

#### Coculture of GSC with CCO

Forty to 100 days after their initiation, CCOs were individually transferred into 1 well of 24-well plate (Falcon Cat#353047). Dissociated EGFP/Luciferase-expressing stable GSCs (2 x 10^4^ cells) were added to each well in 1mL of GSC medium without growth factor. Plates were incubated on orbital agitator (85 RPM). After 2 days, CCOs were washed in PBS and transferred into new 24 well conventional plate. Then, CCO/GSC were maintained under orbital shaking (85 RPM) in cerebral organoid differentiation medium without growth factor with biweekly renewal.

#### Irradiation

Irradiations were performed with a GSR D1 irradiator (GSM) with four sources of Cs-137, with a total activity of around 180 TBq in March 2014, emitting 662 KeV gamma rays. Samples were irradiated at one single dose of 2 Gy (∼1 Gy/min) in 24-well plates. Prior to irradiation, dosimetry was performed in the same irradiation conditions with a cylindrical ionizing chamber 31010 by PTW as the recommendation of the AAPM’S TG-61. This ionizing chamber has a cavity of 0.125 cm3 calibrated in Cs-137 Kerma air with the PTB reference facility number 230451301. The polarity and the ion recombination were measured for this Cs-137 source. Each measurement was corrected using the KTP factor to account for variations in temperature and atmospheric pressure.

#### Luciferase assay in CCO

Pre-warmed medium containing D-luciferin (PerkinElmer Cat#122799) was added to each well to reach a final concentration of 250 μg/mL and immediately processed for monitoring with a Bioluminescence Imager (PerkinElmer IVIS LUMINA II). The flux (photons/sec) was measured each 2 min for 8 min, and the maximum value was recorded.

#### Quantitative PCR (qPCR)

Cells were sorted into tubes containing RLT lysis buffer (Qiagen Cat#79216) and total RNAs were isolated with the RNeasy Micro Kit with DNase treatment (Qiagen Cat#74004) or alternatively with the NucleoSpin RNA XS (Marcherey-Nagel Cat#740902). For qRT-PCR experiments, total RNAs were reverse-transcribed into cDNA using the Reverse Transcription High Capacity Master Mix (Applied Biosystems Cat#4368814). q-PCR was performed on an Applied Biosystems StepOne Real-Time PCR System (RRID:SCR_023455) using Power SYBR GREEN (Applied Biosystems Cat#4367659) with specific primers listed in Table S1. Data were analyzed on StepOne Software (RRID:SCR_014281).

#### Manual and automated multiplex immunofluorescences

CCOs were fixed in 4% paraformaldehyde 3–4 h at room temperature and conserved at 4°C in PBS with 0.2% azide. For 5μm slides, fixed CCOs were embedded in paraffin with Tissue-Tek (Sakura) and sliced with microtome (RM2125RT, Leica Microsystems), and were mounted on SuperFrost Plus (Epredia Cat#J1800AMNZ). After paraffin removal and citrate treatment, slices were blocked 1 h at room temperature in PBS with 10% FCS and 5% BSA (Sigma Aldrich Cat#A7906). The slides were incubated overnight with primary antibodies at 4°C. After PBS washing, the slides were incubated with secondary antibodies (1/1000) at room temperature for 3 h then washed with PBS. Alternatively, 4-plex was performed by automated immunostainnings on a Roche Ventana BenchMark ULTRA IHC/ISH System (RRID:SCR_025506) according to the manufacturer’s protocol. Primary antibodies were the same as above and OmniMap anti-Ms HRP and anti-Rb HRP (Roche Cat#5269652001 and Cat#5269679001) secondary antibodies were coupled to OPAL520 (AKOYA Biosciences Cat# FP1487001KT), OPAL570 (AKOYA Biosciences Cat#FP1488001KT), OPAL620 (AKOYA Biosciences Cat#FP1495001KT), OPAL650 (AKOYA Biosciences Cat#FP1496001KT) reagents with Tyramide Signal Amplification. Slides were mounted with Fluoromount-DAPI (Southern Biotechnologies Cat#0100-20). Images were captured on a Leica SP8 LIGHTNING confocal microscope (RRID:SCR_018169) with the following objectives: ×20/0.75 NA, and ×63/1.3 NA Oil. Images were analyzed on Leica Application Suite X (RRID:SCR_013673) or IMARIS (RRID:SCR_007370) softwares.

#### 3D Immunofluorescence and CE3D clearing

Fixed CCOs were embedded in 2% low melting point agarose in PBS and sliced at 500μm with a Leica VT1200S vibrating microtome (RRID:SCR_020243). Slices were stained and cleared following the Ce3D protocol with modifications [28]. Briefly, slices were blocked 24h with Alternative Blocking Buffer (ABB) (PBS-TABS [Sigma Aldrich Cat#18912] with 0.3% Triton X-100 [Sigma Aldrich Cat#T8787], 1% BSA, 1X BD Perm/Wash Buffer [BD Cat#554723], alternatively 1% of Donkey Serum [Eurobio Scientific Cat#CAEMTN00-0U] was added for secondary antibodies). Slices were incubated 72h with primary antibodies in ABB at 37°C under 85 RPM. After a 12 h washing with Conventional Washing Buffer (CWB) (PBS-TABS with 0.3% Triton X-100, 0.5% 1-thioglycerol [Sigma Aldrich Cat#M1753], 0.1% Saponine [Sigma Aldrich Cat#S-4521]) at 37°C under 85 RPM, slices were incubated 72h with secondary antibodies (1/1000) and with 5μg/mL of DAPI (Sigma Aldrich Cat#D9542) in ABB at 37°C under 85 RPM. After a washing with CWB, slices were placed into 24 well plate glass bottom (Azenta Cat#4TI-0243), and were progressively cleared under orbital shaking (100 RPM) with 30%, 50%, 70%, 90% gradient of Ce3D solution in PBS, each for 1h30 then with 100% Ce3D solution overnight. Supernatant was eliminated and cover slips (Epredia Cat#CB00120RA120MNZ0) were placed. Images were acquired on a Spinning-Disk confocal microscope (Nikon, GATTACA) with a 1mm piezoelectric (Piezoconcept) with the following objectives: ×10/0.45 NA, ×20/0.75 NA, ×40/0.45 NA, and ×60/1.4 NA oil. Images were analyzed with IMARIS software (RRID:SCR_007370).

### Quantification and statistical analysis

Each data point in figures represents a single organoid with at least two different batches per figure. All data points were plotted, and the median along with the interquartile range (1st and 3rd quartiles) are shown. One-way ANOVA was performed using Kruskal–Wallis test with Dunn’s post hoc correction for multiple comparisons (*p* < 0.05: ∗; *p* < 0.01: ∗∗), or Mann–Whitney test for pairwise comparisons (*p* < 0.05: #; *p* < 0.01: ##), using GraphPad Prism 9 (RRID:SCR_002798). For flow cytometry analyses, each data point corresponds to a single CO/CCO. Few CCOs had a low CD45-positive cell content (less than 0.040%) and/or contained less than 20% P2RY12-positive cells within the CD45^+^ population; they were therefore removed from the analyses except in Figure 5.

## Supplemental information

Document S1. Figures S1–S12 and Table S1

Document S2. Article plus supplemental information

## References

[1] Bao S., Wu Q., McLendon R.E., Hao Y., Shi Q., Hjelmeland A.B., Dewhirst M.W., Bigner D.D., Rich J.N. (2006). Glioma stem cells promote radioresistance by preferential activation of the DNA damage response. Nature.

[2] Vollmann-Zwerenz A., Leidgens V., Feliciello G., Klein C.A., Hau P. (2020). Tumor Cell Invasion in Glioblastoma. Int. J. Mol. Sci..

[3] Read R.D., Tapp Z.M., Rajappa P., Hambardzumyan D. (2024). Glioblastoma microenvironment-from biology to therapy. Genes Dev..

[4] Jeon H.-M., Kim J.-Y., Cho H.J., Lee W.J., Nguyen D., Kim S.S., Oh Y.T., Kim H.-J., Jung C.-W., Pinero G. (2023). Tissue factor is a critical regulator of radiation therapy-induced glioblastoma remodeling. Cancer Cell.

[5] Gutmann D.H., Kettenmann H. (2019). Microglia/Brain Macrophages as Central Drivers of Brain Tumor Pathobiology. Neuron.

[6] Akkari L., Bowman R.L., Tessier J., Klemm F., Handgraaf S.M., de Groot M., Quail D.F., Tillard L., Gadiot J., Huse J.T. (2020). Dynamic changes in glioma macrophage populations after radiotherapy reveal CSF-1R inhibition as a strategy to overcome resistance. Sci. Transl. Med..

[7] Pombo Antunes A.R., Scheyltjens I., Lodi F., Messiaen J., Antoranz A., Duerinck J., Kancheva D., Martens L., De Vlaminck K., Van Hove H. (2021). Single-cell profiling of myeloid cells in glioblastoma across species and disease stage reveals macrophage competition and specialization. Nat. Neurosci..

[8] Amankulor N.M., Kim Y., Arora S., Kargl J., Szulzewsky F., Hanke M., Margineantu D.H., Rao A., Bolouri H., Delrow J. (2017). Mutant IDH1 regulates the tumor-associated immune system in gliomas. Genes Dev..

[9] Rosińska S., Gavard J. (2021). Tumor Vessels Fuel the Fire in Glioblastoma. Int. J. Mol. Sci..

[10] Wang W., Li T., Cheng Y., Li F., Qi S., Mao M., Wu J., Liu Q., Zhang X., Li X. (2024). Identification of hypoxic macrophages in glioblastoma with therapeutic potential for vasculature normalization. Cancer Cell.

[11] Chiaradia I., Lancaster M.A. (2020). Brain organoids for the study of human neurobiology at the interface of in vitro and in vivo. Nat. Neurosci..

[12] Krieger T.G., Tirier S.M., Park J., Jechow K., Eisemann T., Peterziel H., Angel P., Eils R., Conrad C. (2020). Modeling glioblastoma invasion using human brain organoids and single-cell transcriptomics. Neuro Oncol..

[13] Linkous A., Balamatsias D., Snuderl M., Edwards L., Miyaguchi K., Milner T., Reich B., Cohen-Gould L., Storaska A., Nakayama Y. (2019). Modeling Patient-Derived Glioblastoma with Cerebral Organoids. Cell Rep..

[14] Cakir B., Xiang Y., Tanaka Y., Kural M.H., Parent M., Kang Y.-J., Chapeton K., Patterson B., Yuan Y., He C.-S. (2019). Engineering of human brain organoids with a functional vascular-like system. Nat. Methods.

[15] Mansour A.A., Gonçalves J.T., Bloyd C.W., Li H., Fernandes S., Quang D., Johnston S., Parylak S.L., Jin X., Gage F.H. (2018). An in vivo model of functional and vascularized human brain organoids. Nat. Biotechnol..

[16] Zhang W., Jiang J., Xu Z., Yan H., Tang B., Liu C., Chen C., Meng Q. (2023). Microglia-containing human brain organoids for the study of brain development and pathology. Mol. Psychiatry.

[17] Park D.S., Kozaki T., Tiwari S.K., Moreira M., Khalilnezhad A., Torta F., Olivié N., Thiam C.H., Liani O., Silvin A. (2023). iPS-cell-derived microglia promote brain organoid maturation via cholesterol transfer. Nature.

[18] Schafer S.T., Mansour A.A., Schlachetzki J.C.M., Pena M., Ghassemzadeh S., Mitchell L., Mar A., Quang D., Stumpf S., Ortiz I.S. (2023). An in vivo neuroimmune organoid model to study human microglia phenotypes. Cell.

[19] Sun X.-Y., Ju X.-C., Li Y., Zeng P.-M., Wu J., Zhou Y.-Y., Shen L.-B., Dong J., Chen Y.-J., Luo Z.-G. (2022). Generation of vascularized brain organoids to study neurovascular interactions. eLife.

[20] Marcelo K.L., Goldie L.C., Hirschi K.K. (2013). Regulation of endothelial cell differentiation and specification. Circ. Res..

[21] Ginhoux F., Greter M., Leboeuf M., Nandi S., See P., Gokhan S., Mehler M.F., Conway S.J., Ng L.G., Stanley E.R. (2010). Fate mapping analysis reveals that adult microglia derive from primitive macrophages. Science.

[22] Atkins M.H., Scarfò R., McGrath K.E., Yang D., Palis J., Ditadi A., Keller G.M. (2021). Modeling human yolk sac hematopoiesis with pluripotent stem cells. J. Exp. Med..

[23] Vargas-Valderrama A., Ponsen A.-C., Le Gall M., Clay D., Jacques S., Manoliu T., Rouffiac V., Ser-le-Roux K., Quivoron C., Louache F. (2022). Endothelial and hematopoietic hPSCs differentiation via a hematoendothelial progenitor. Stem Cell Res. Ther..

[24] Azzoni E., Conti V., Campana L., Dellavalle A., Adams R.H., Cossu G., Brunelli S. (2014). Hemogenic endothelium generates mesoangioblasts that contribute to several mesodermal lineages in vivo. Development.

[25] Lancaster M.A., Renner M., Martin C.-A., Wenzel D., Bicknell L.S., Hurles M.E., Homfray T., Penninger J.M., Jackson A.P., Knoblich J.A. (2013). Cerebral organoids model human brain development and microcephaly. Nature.

[26] El-Habr E.A., Dubois L.G., Burel-Vandenbos F., Bogeas A., Lipecka J., Turchi L., Lejeune F.-X., Coehlo P.L.C., Yamaki T., Wittmann B.M. (2017). A driver role for GABA metabolism in controlling stem and proliferative cell state through GHB production in glioma. Acta Neuropathol..

[27] Xie Y., He L., Lugano R., Zhang Y., Cao H., He Q., Chao M., Liu B., Cao Q., Wang J. (2021). Key molecular alterations in endothelial cells in human glioblastoma uncovered through single-cell RNA sequencing. JCI Insight.

[28] Gauthier L.R., Saati M., Bensalah-Pigeon H., Ben M’Barek K., Gitton-Quent O., Bertrand R., Busso D., Mouthon M.-A., Collura A., Junier M.-P. (2020). The HIF1α/JMY pathway promotes glioblastoma stem-like cell invasiveness after irradiation. Sci. Rep..

[29] Wu L., Wu W., Zhang J., Zhao Z., Li L., Zhu M., Wu M., Wu F., Zhou F., Du Y. (2022). Natural Coevolution of Tumor and Immunoenvironment in Glioblastoma. Cancer Discov..

[30] Daneman R., Prat A. (2015). The Blood–Brain Barrier. Cold Spring Harb. Perspect. Biol..

[31] Carmeliet P., Tessier-Lavigne M. (2005). Common mechanisms of nerve and blood vessel wiring. Nature.

[32] Dao L., You Z., Lu L., Xu T., Sarkar A.K., Zhu H., Liu M., Calandrelli R., Yoshida G., Lin P. (2024). Modeling blood-brain barrier formation and cerebral cavernous malformations in human PSC-derived organoids. Cell Stem Cell.

[33] Quintard C., Tubbs E., Jonsson G., Jiao J., Wang J., Werschler N., Laporte C., Pitaval A., Bah T.-S., Pomeranz G. (2024). A microfluidic platform integrating functional vascularized organoids-on-chip. Nat. Commun..

[34] Paşca A.M., Sloan S.A., Clarke L.E., Tian Y., Makinson C.D., Huber N., Kim C.H., Park J.-Y., O’Rourke N.A., Nguyen K.D. (2015). Functional cortical neurons and astrocytes from human pluripotent stem cells in 3D culture. Nat. Methods.

[35] Renner M., Lancaster M.A., Bian S., Choi H., Ku T., Peer A., Chung K., Knoblich J.A. (2017). Self-organized developmental patterning and differentiation in cerebral organoids. EMBO J..

[36] Thion M.S., Ginhoux F., Garel S. (2018). Microglia and early brain development: An intimate journey. Science.

[37] Gosselin D., Skola D., Coufal N.G., Holtman I.R., Schlachetzki J.C.M., Sajti E., Jaeger B.N., O’Connor C., Fitzpatrick C., Pasillas M.P. (2017). An environment-dependent transcriptional network specifies human microglia identity. Science.

[38] Mittelbronn M., Dietz K., Schluesener H.J., Meyermann R. (2001). Local distribution of microglia in the normal adult human central nervous system differs by up to one order of magnitude. Acta Neuropathol..

[39] Sinnaeve J., Mobley B.C., Ihrie R.A. (2018). Space Invaders: Brain Tumor Exploitation of the Stem Cell Niche. Am. J. Pathol..

[40] Kusne Y., Sanai N. (2015). The SVZ and Its Relationship to Stem Cell Based Neuro-oncogenesis. Adv. Exp. Med. Biol..

[41] Stafford J.H., Hirai T., Deng L., Chernikova S.B., Urata K., West B.L., Brown J.M. (2016). Colony stimulating factor 1 receptor inhibition delays recurrence of glioblastoma after radiation by altering myeloid cell recruitment and polarization. Neuro Oncol..

[42] Maas S.L.N., Abels E.R., Van De Haar L.L., Zhang X., Morsett L., Sil S., Guedes J., Sen P., Prabhakar S., Hickman S.E. (2020). Glioblastoma hijacks microglial gene expression to support tumor growth. J. Neuroinflammation.

[43] Blériot C., Dunsmore G., Alonso-Curbelo D., Ginhoux F. (2024). A temporal perspective for tumor-associated macrophage identities and functions. Cancer Cell.

[44] Hara T., Chanoch-Myers R., Mathewson N.D., Myskiw C., Atta L., Bussema L., Eichhorn S.W., Greenwald A.C., Kinker G.S., Rodman C. (2021). Interactions between cancer cells and immune cells drive transitions to mesenchymal-like states in glioblastoma. Cancer Cell.

[45] Zhou W., Ke S.Q., Huang Z., Flavahan W., Fang X., Paul J., Wu L., Sloan A.E., McLendon R.E., Li X. (2015). Periostin secreted by glioblastoma stem cells recruits M2 tumour-associated macrophages and promotes malignant growth. Nat. Cell Biol..

[46] Jhaveri N., Chen T.C., Hofman F.M. (2016). Tumor vasculature and glioma stem cells: Contributions to glioma progression. Cancer Lett..

[47] Marhuenda E., Fabre C., Zhang C., Martin-Fernandez M., Iskratsch T., Saleh A., Bauchet L., Cambedouzou J., Hugnot J.-P., Duffau H. (2021). Glioma stem cells invasive phenotype at optimal stiffness is driven by MGAT5 dependent mechanosensing. J. Exp. Clin. Cancer Res..

[48] Cuddapah V.A., Robel S., Watkins S., Sontheimer H. (2014). A neurocentric perspective on glioma invasion. Nat. Rev. Neurosci..

[49] Watson S.S., Duc B., Kang Z., de Tonnac A., Eling N., Font L., Whitmarsh T., Massara M., Bodenmiller B., Hausser J. (2024). Microenvironmental reorganization in brain tumors following radiotherapy and recurrence revealed by hyperplexed immunofluorescence imaging. Nat. Commun..

[50] Voshart D.C., Oshima T., Jiang Y., van der Linden G.P., Ainslie A.P., Reali Nazario L., van Buuren-Broek F., Scholma A.C., van Weering H.R.J., Brouwer N. (2024). Radiotherapy induces persistent innate immune reprogramming of microglia into a primed state. Cell Rep..

[51] Wang L., Dou X., Chen S., Yu X., Huang X., Zhang L., Chen Y., Wang J., Yang K., Bugno J. (2023). YTHDF2 inhibition potentiates radiotherapy antitumor efficacy. Cancer Cell.

[52] Antonucci L., Canciani G., Mastronuzzi A., Carai A., Del Baldo G., Del Bufalo F. (2022). CAR-T Therapy for Pediatric High-Grade Gliomas: Peculiarities, Current Investigations and Future Strategies. Front. Immunol..

[53] Golebiewska A., Hau A.-C., Oudin A., Stieber D., Yabo Y.A., Baus V., Barthelemy V., Klein E., Bougnaud S., Keunen O. (2020). Patient-derived organoids and orthotopic xenografts of primary and recurrent gliomas represent relevant patient avatars for precision oncology. Acta Neuropathol..

[54] Jacob F., Salinas R.D., Zhang D.Y., Nguyen P.T.T., Schnoll J.G., Wong S.Z.H., Thokala R., Sheikh S., Saxena D., Prokop S. (2020). A Patient-Derived Glioblastoma Organoid Model and Biobank Recapitulates Inter- and Intra-tumoral Heterogeneity. Cell.

[55] Logun M., Wang X., Sun Y., Bagley S.J., Li N., Desai A., Zhang D.Y., Nasrallah M.P., Pai E.L.-L., Oner B.S. (2025). Patient-derived glioblastoma organoids as real-time avatars for assessing responses to clinical CAR-T cell therapy. Cell Stem Cell.

[56] Rajan R.G., Fernandez-Vega V., Sperry J., Nakashima J., Do L.H., Andrews W., Boca S., Islam R., Chowdhary S.A., Seldin J. (2023). In Vitro and In Vivo Drug-Response Profiling Using Patient-Derived High-Grade Glioma. Cancers (Basel).

[57] Soubéran A., Jiguet-Jiglaire C., Toutain S., Morando P., Baeza-Kallee N., Appay R., Boucard C., Graillon T., Meyer M., Farah K. (2025). Brain tumoroids: Treatment prediction and drug development for brain tumors with fast, reproducible, and easy-to-use personalized models. Neuro Oncol..

[58] Fedorova V., Pospisilova V., Vanova T., Amruz Cerna K., Abaffy P., Sedmik J., Raska J., Vochyanova S., Matusova Z., Houserova J. (2023). Glioblastoma and cerebral organoids: development and analysis of an in vitro model for glioblastoma migration. Mol. Oncol..

[59] Mangena V., Chanoch-Myers R., Sartore R., Paulsen B., Gritsch S., Weisman H., Hara T., Breakefield X.O., Breyne K., Regev A. (2025). Glioblastoma Cortical Organoids Recapitulate Cell-State Heterogeneity and Intercellular Transfer. Cancer Discov..

[60] Sarnow K., Majercak E., Qurbonov Q., Cruzeiro G.A.V., Jeong D., Haque I.A., Khalil A., Baird L.C., Filbin M.G., Tang X. (2025). Neuroimmune-competent human brain organoid model of diffuse midline glioma. Neuro Oncol..

[61] Baisiwala S., Fazzari E., Li M.X., Martija A., Azizad D.J., Sun L., Herrera G., Phan T., Monteleone A., Kan R.L. (2026). A human tumor-immune organoid model of glioblastoma. Cell Rep..

[62] Silvestre D.C., Pineda J.R., Hoffschir F., Studler J.M., Mouthon M.A., Pflumio F., Junier M.P., Chneiweiss H., Boussin F.D. (2011). Alternative lengthening of telomeres in human glioma stem cells. Stem Cell..

