# Supplementary material for: Human cerebral organoids with microglia and vasculature model glioma stem cell interactions and radiotherapy response
Source: Cell Rep Methods. 2026 May 5;6(6):101425. doi: 10.1016/j.crmeth.2026.101425 (PMC13282650; doi:10.1016/j.crmeth.2026.101425)
Supplement: Document S1. Figures S1–S12 and Table S1 [file mmc1.pdf]

Cell Reports Methods, Volume *6*

Supplemental information

Human cerebral organoids with microglia and vasculature model glioma stem cell interactions and radiotherapy response

Jérémy Raguin, Noa Legrand, Thierry Kortulewski, Oriane Bergiers, Christine Granotier-Beckers, Laure Chatrousse, Alexandra Benchoua, Laurent R. Gauthier, François D. Boussin, and Marc-André Mouthon

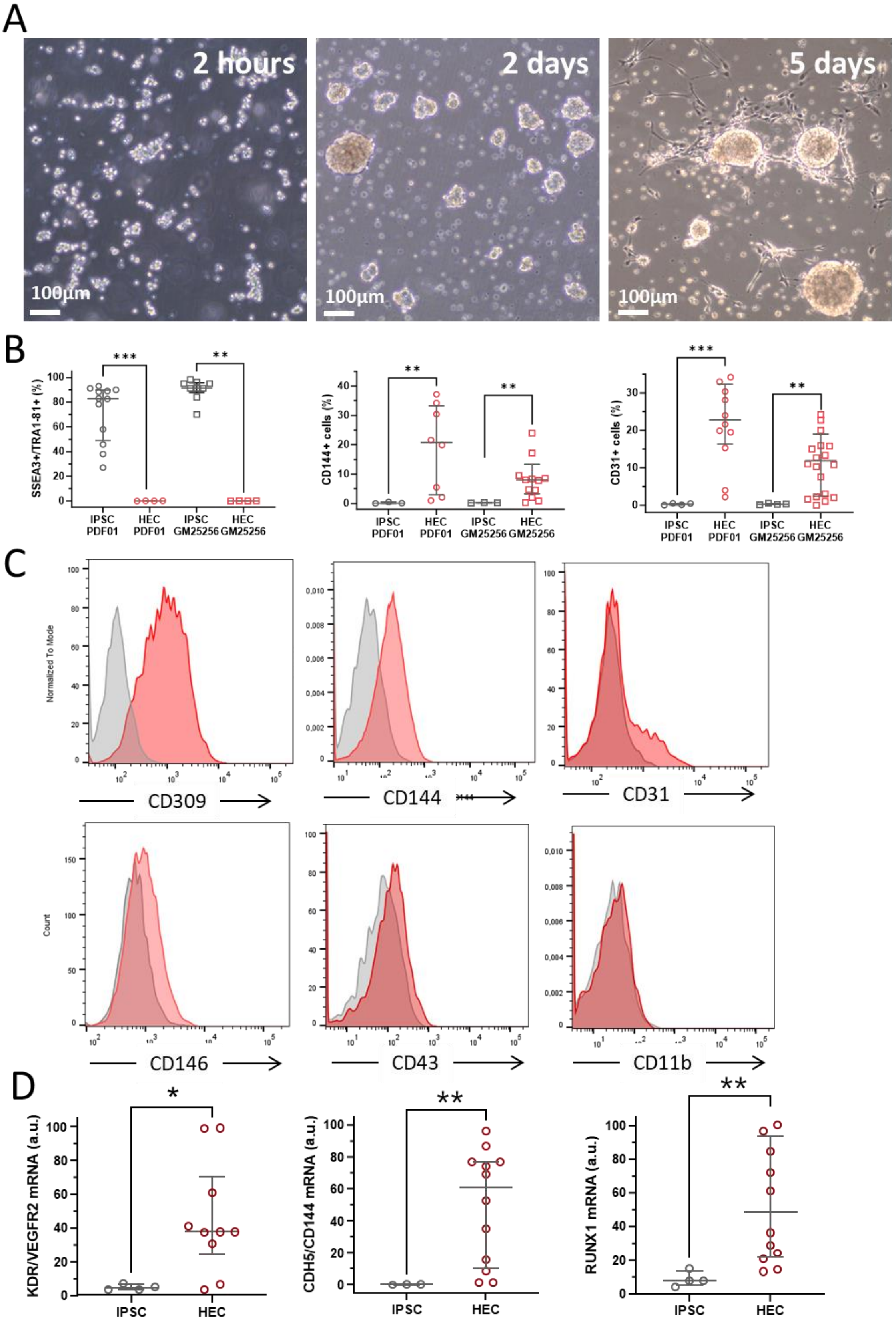

Fig. S1: Differentiation of iPSC into HECs. Related to STAR Methods.

(A) Phase contrast representations of the early steps of PDF01 iPSC differentiation into HECs. Clones begun to adhere around day 5. FACS analyses shows the loss of IPSC markers SSEA3/TRA1-81 (B) on HECs and the appearance of HEC markers CD144, CD309, CD31 and CD146 (C; derived from PDF01). (D) Expression by RT-qPCR of a panel of genes shows the differentiation in HECs derived from GM25256 iPSC line. Data are presented as median (interquartile range); each dot represents a single experiment. Groups were compared pairwise using the Mann–Whitney test (**: p<0.01, ***: p<0.005).

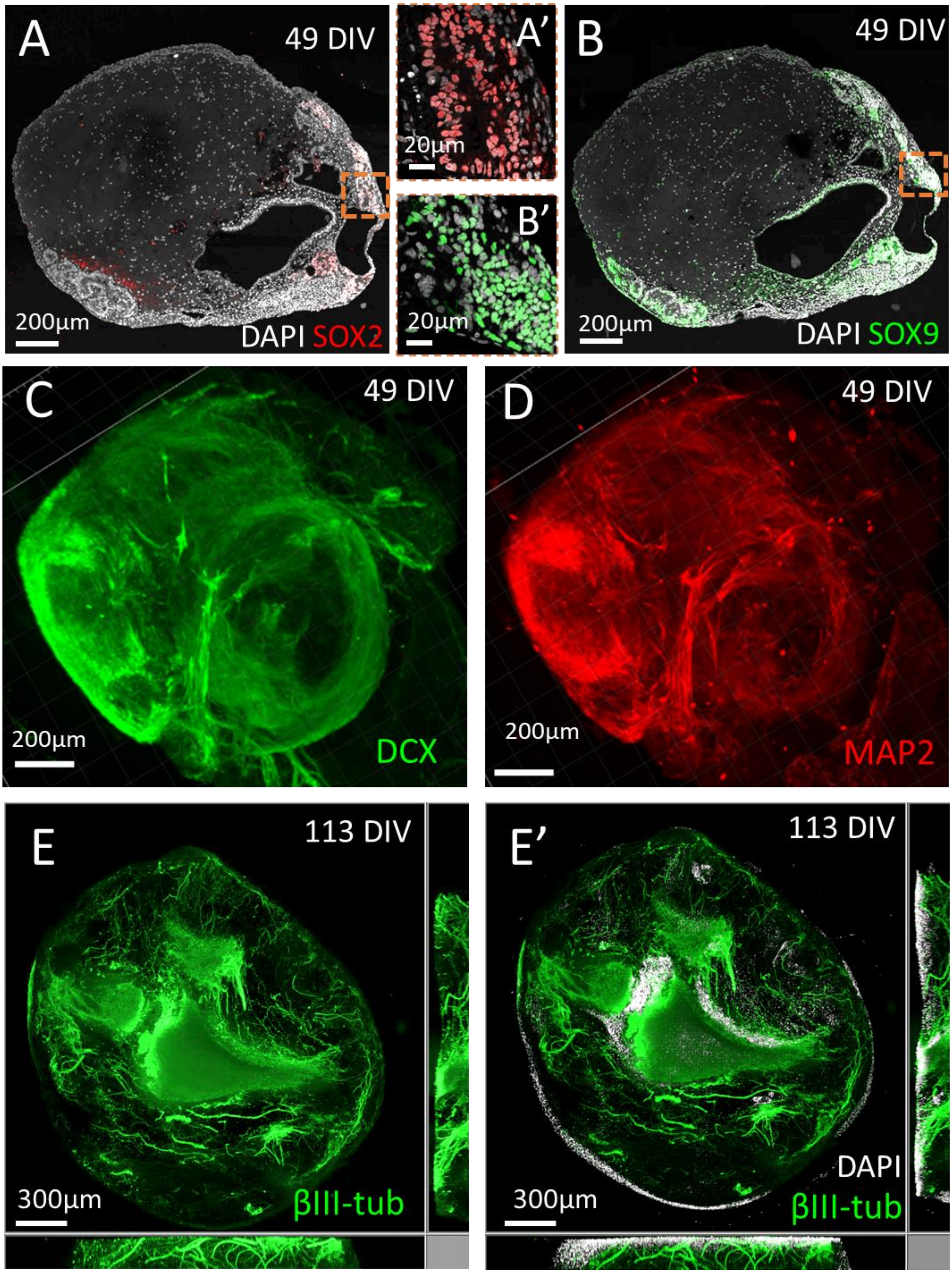

Fig. S2: Neural/glial progenitors, neurons and astrocytes are contained in CCO. Related to Figure 1.

Immunostaining for progenitors (SOX2, SOX9) and neurons (MAP2, β3 tubulin and DCX) were performed at 49 to 126 days after CCO initiation on 5µm sections (A, B) or on 500 µm sections (C, D and E). CCOs were derived from PDF01 (A-D) and GM25256 (E-E'') iPSC lines.

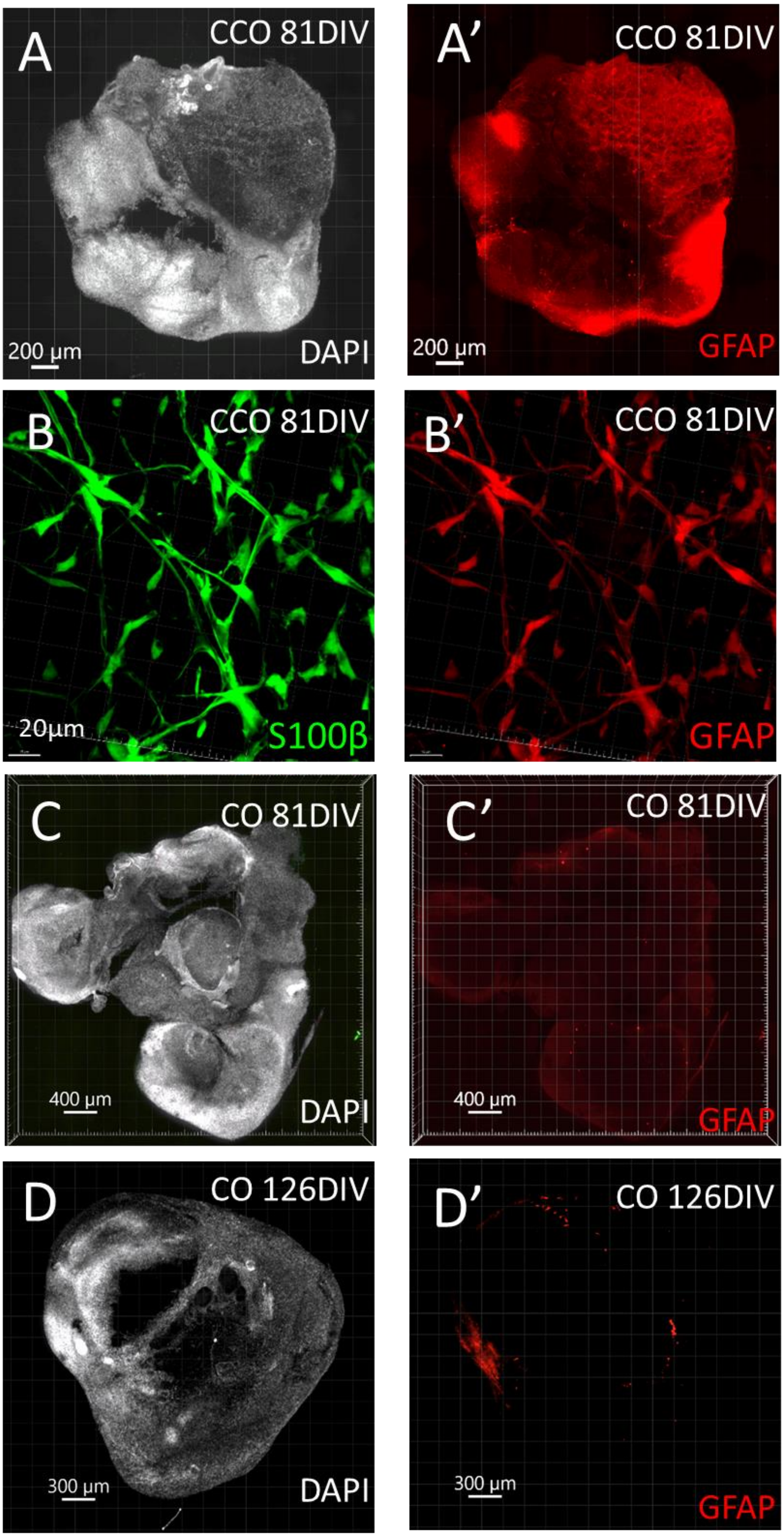

Fig S3: Astrocytes appear earlier in CCOs than in COs. Related to Figure 1.

The presence of astrocytes was examined by GFAP/S100β immunostaining on 500µm thick sections of CCOs (A-B) and COs (C-D) at 81 and 126 after their initiation. CCOs were derived from GM25256 iPSCs.

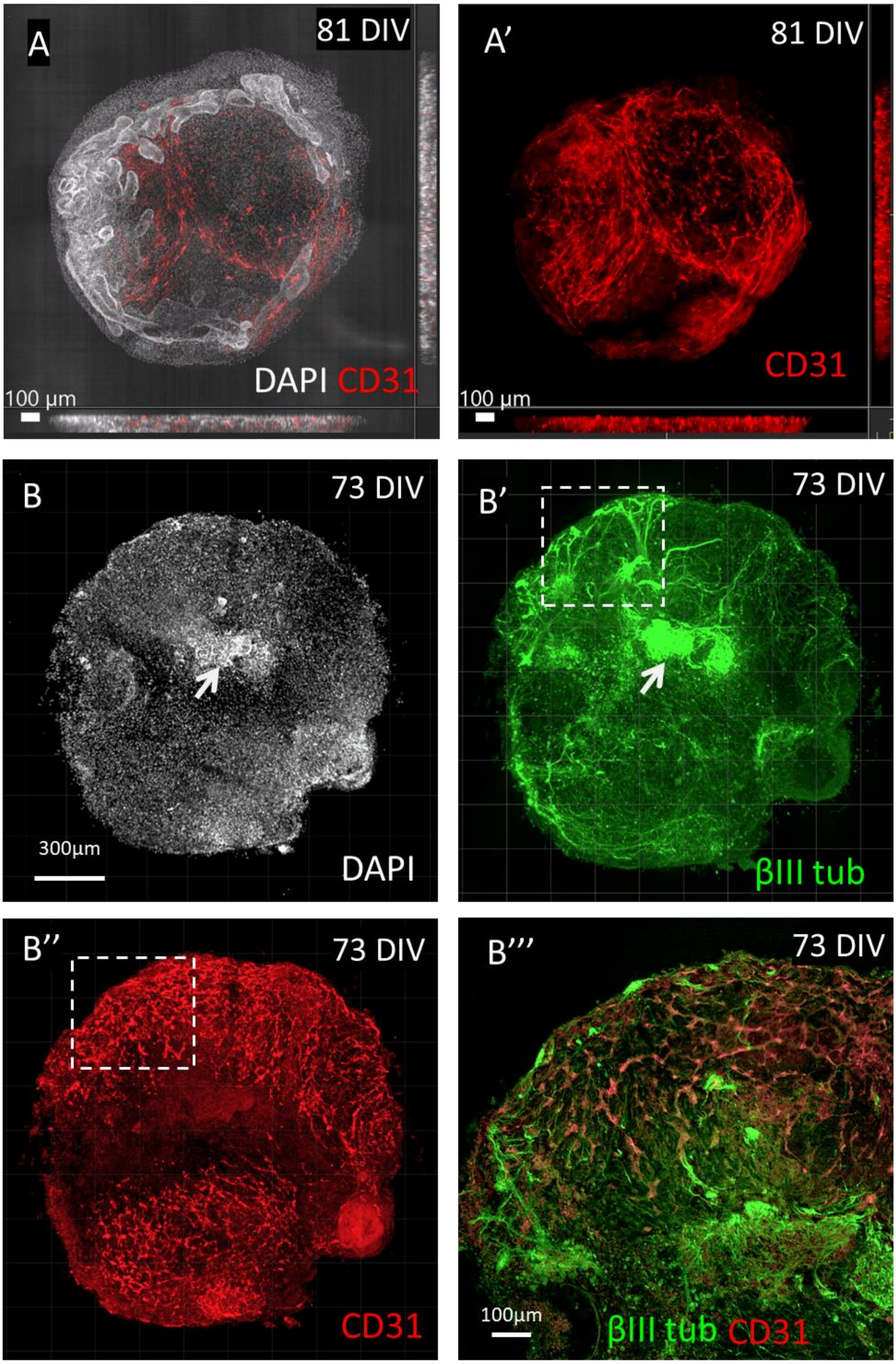

Fig. S4: Vascular-like structures developed in the inner regions of CCOs and were intermingled with β3-tubulin–positive neurons. Related to Figure 1.

Immunofluorescence staining for CD31 and βIII-tubulin were performed on 400µm thick CCO sections. (A’) X-Y projections of a section reveal the presence of the CD31 staining in the inner region. A magnified view of the merged immunostaining shown in B’ and B’’ is presented in B’’’. Regions with dense nuclei are stained for βIII-tubulin (arrow). CCOs were derived from GM25256 iPSCs.

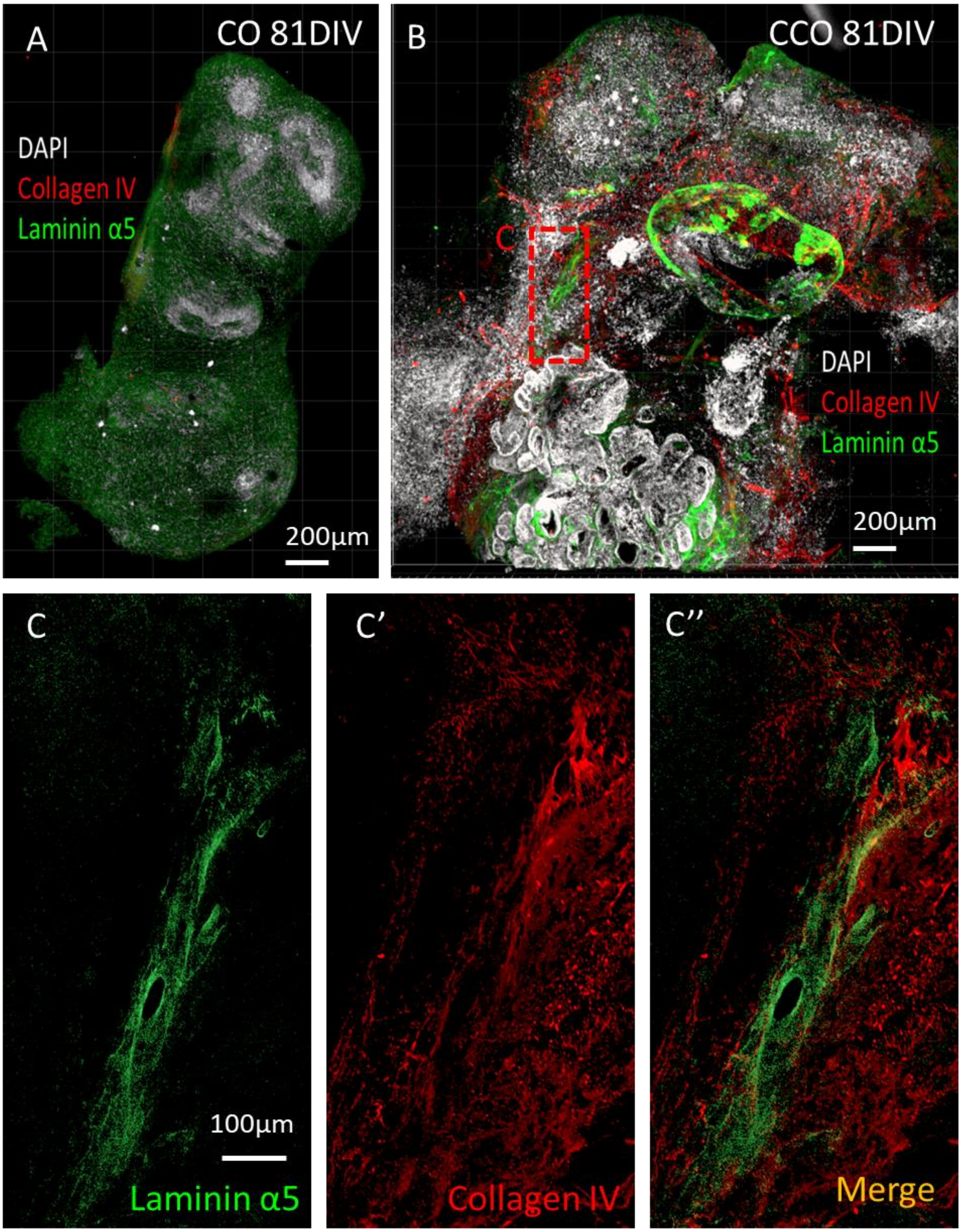

Fig. S5: Organoid-secreted laminin-α5 and collagen IV were detected in CCOs but not in COs. Related to Figure 1.

Immunostaining for human laminin-α5 and collagen IV on 400µm thick sections reveals their absence in COs (A) but were clearly detected in CCOs (B). A magnified view of the CCO immunostaining is shown in panels C–C'' and shows that Col IV and laminin-α5 localize to the same vascular-like structures. CCOs were derived from GM25256 iPSCs.

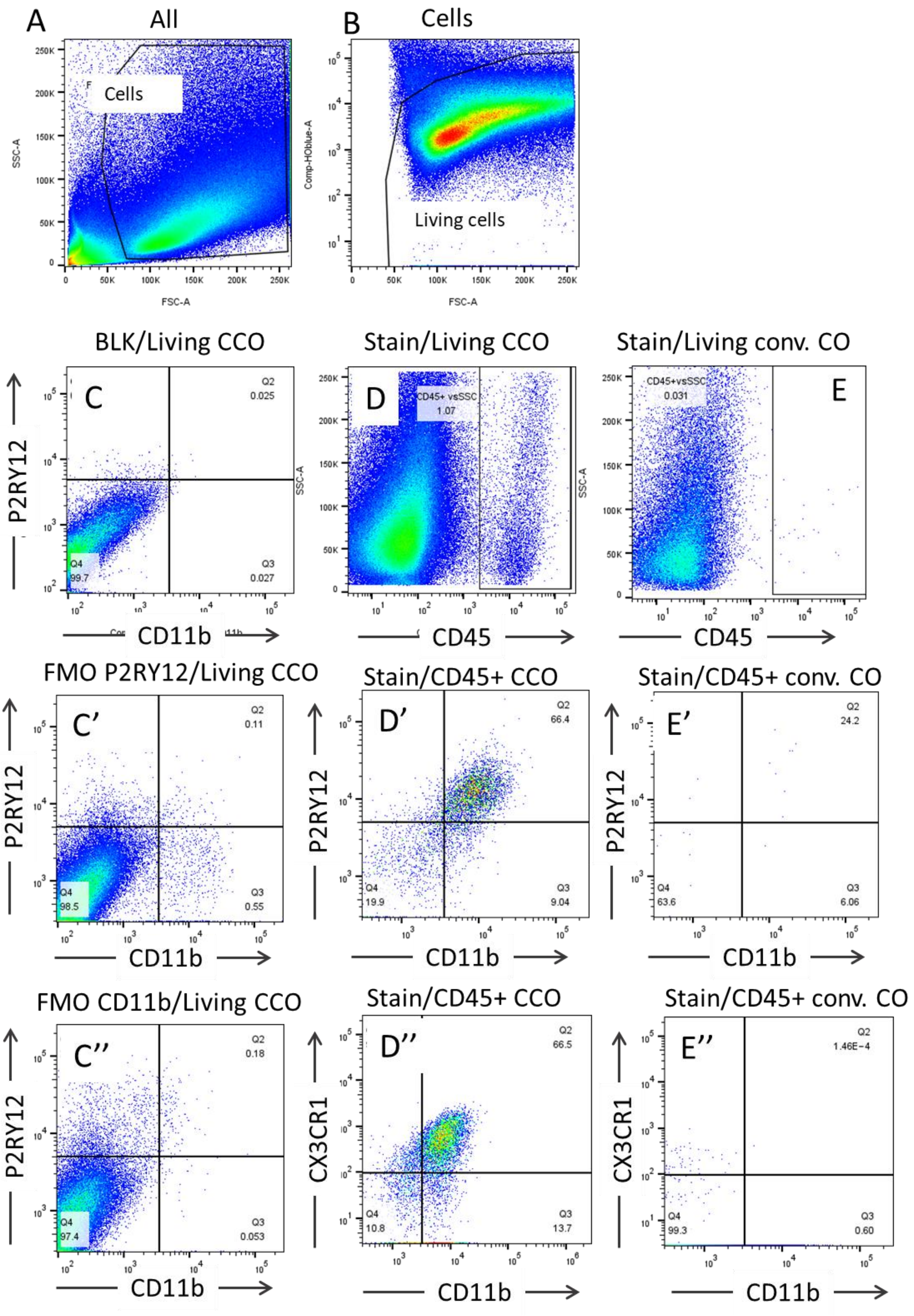

Fig. S6: Characterization of microglial-like cells by FACS. Related to Figure 5.

Single cell suspensions were obtained from digestion of CCOs (A, B, D-D'') and classical COs (E-E'') at 60-80 DIV and analysed by FACS. The gating strategy for identification of iMG in CCO is shown. Gates were set according to unstained cells (C) and Fluorescent Minus One (FMO) controls (C'-C''). iMG are contained in CD45+ population and expressed CD11b, P2RY12 and CX3CR1. CCOs were derived from GM25256 iPSCs.

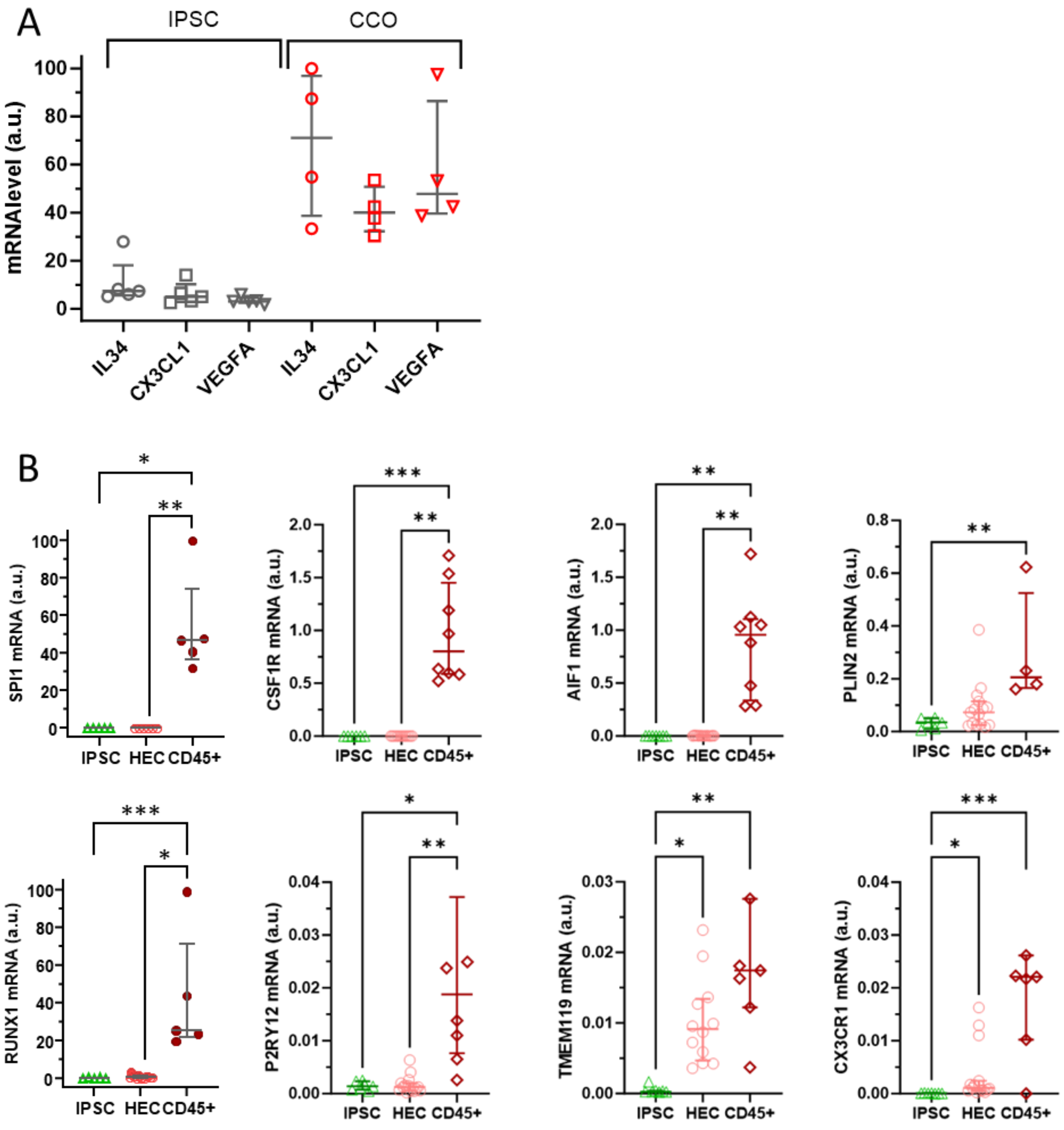

Fig. S7: Analysis of the expression of multiple genes by RT-qPCR in iPSCs, CCOs, and in CD45⁺ cells sorted from CCOs. Related to Figure 5.

(A) Expression of IL34, CXCL1 and VEGFA was determined by RT-qPCR in CCOs. (B) Expression of microglial genes was determined by RT-qPCR in sorted CD45+CD11b+ iMG and compared to parental iPSC and to HECs. mRNA expressions were normalized to 18S, a.u.: arbitrary unit. Data are presented as median with interquartile range; each dot represents an individual experiment. CCOs were derived from GM25256 iPSCs. Comparisons across all groups were performed with the Kruskal–Wallis test with Dunn's post hoc multiple-comparisons test (*: $p<0.05$, **: $p<0.01$, ***: $p<0.005$).

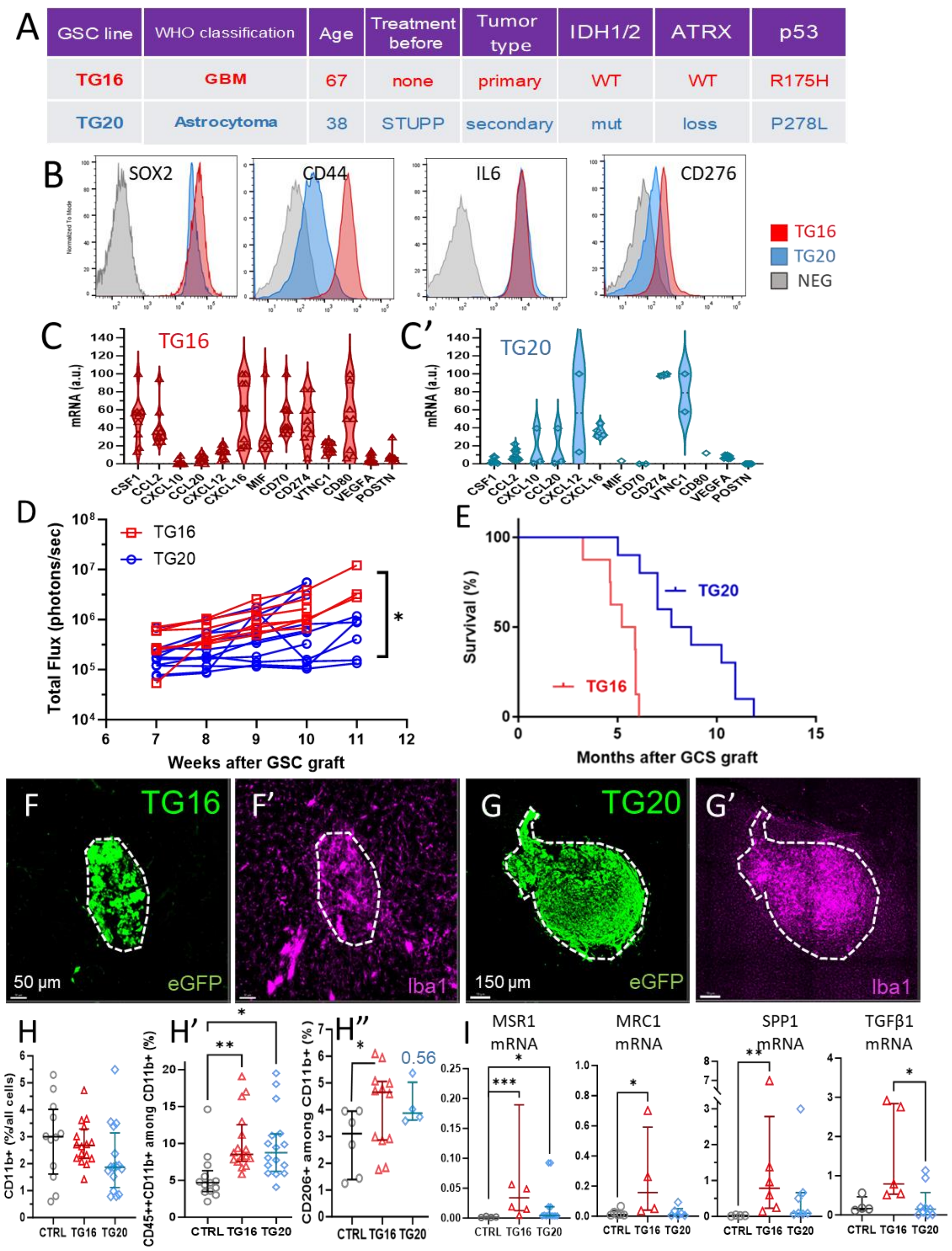

Fig. S8: Characterization of GCS lines and development of brain tumors following xenotransplantation in mice. Related to STAR Methods.

(A) Two GSC lines established from GBM (TG16) and astrocytoma grade IV (TG20) patients were used in this study. (B) They were cultured in neural stem cell medium as gliospheres and they expressed the stem cell marker SOX2, the mesenchymal marker CD44, the cytokine IL-6 and the immune checkpoint CD276. The expression of a panel of cytokines chemokines and immune modulators was analyzed by RT-qPCR (C). TG16 and TG20 lines (10^5 cells) were transplanted into the striatum of Nude mice. (D) Tumor growth was followed by luciferase bioluminescence and longitudinal bioluminescence measurements revealed higher growth of TG16 line compared to TG20, as determined by two-way mixed-effects ANOVA with Geiser–Greenhouse correction. (E) Survival analysis indicated greater aggressiveness in TG16- compared to TG20-transplanted mice. (F-G) Four months after graft, brains were collected and subjected to immunohistologic analyses for EGFP-GSCs, CD31 and Iba1 macrophages. Projection views of 500µm brain sections from TG16 graft and from TG20. (H) Brain tumors were dissected and dissociated then analyzed by FACS for their content in CD11b+ cells (i.e. macrophages and microglial cells). TAM being recruited from blood are contained within the CD45++CD11b+ subpopulation. (I) CD11b+ cells were sorted from tumors and the expression TAM genes was analyzed by RT-qPCR. Data are presented as median with interquartile range; each dot represents an individual mouse. Comparisons across all groups were performed with the Kruskal–Wallis test with Dunn's post hoc multiple-comparisons test (*: p<0.05, **: p<0.01, ***: p<0.005).

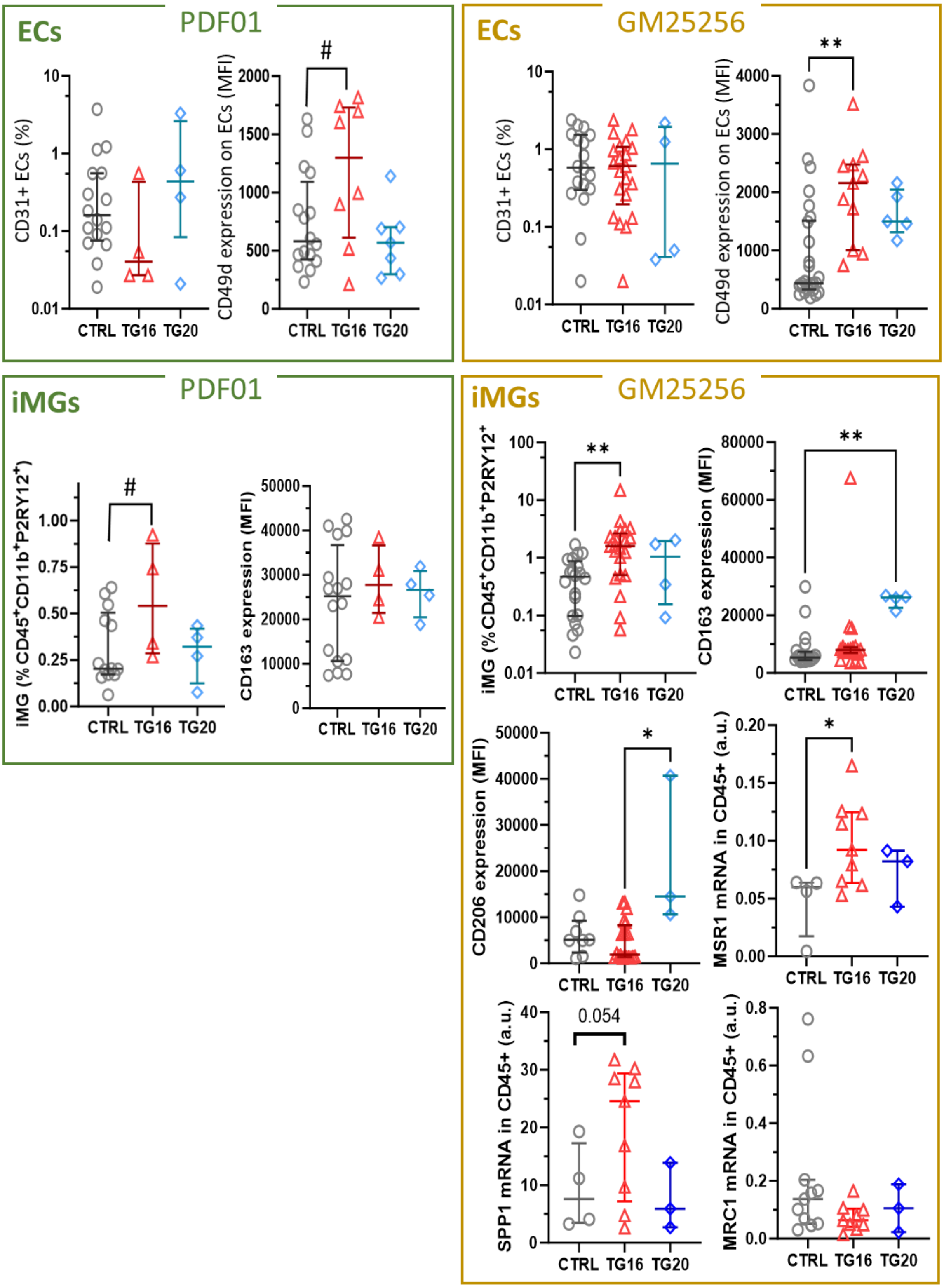

Fig. S9: Endothelial (ECs) and microglial-like cells (iMGs) adopt tumor-associated phenotypes in CCOs cocultured with GSCs. Related to Figure 6.

CCOs obtained from PDF01 (left panel) and GM25256 (right panel) iPSC lines were cocultured with TG16 and TG20 GSCs for four weeks then were dissociated for FACS analyses. (ECs): The percentage of CD31-positive cells and their expression level of CD49d were determined. (iMGs): The percentage CD45+CD11b+P2RY12+ (iMG) and their expression of CD163 and CD206 were determined. The expression of MSR1, SPP1 and MCR1 genes was determined by RT-qPCR in sorted $CD45^{+}$ cells and normalized according to CSF1R (arbitrary unit: a.u.). Data are presented as median with interquartile range; each dot represents an individual CCO/CO. Comparisons across all groups were performed with the Kruskal–Wallis test with Dunn’s post hoc multiple-comparisons test (*: p<0.05, **: p<0.01). Comparisons to CTRL was also performed with Mann–Whitney (#: p<0.05).

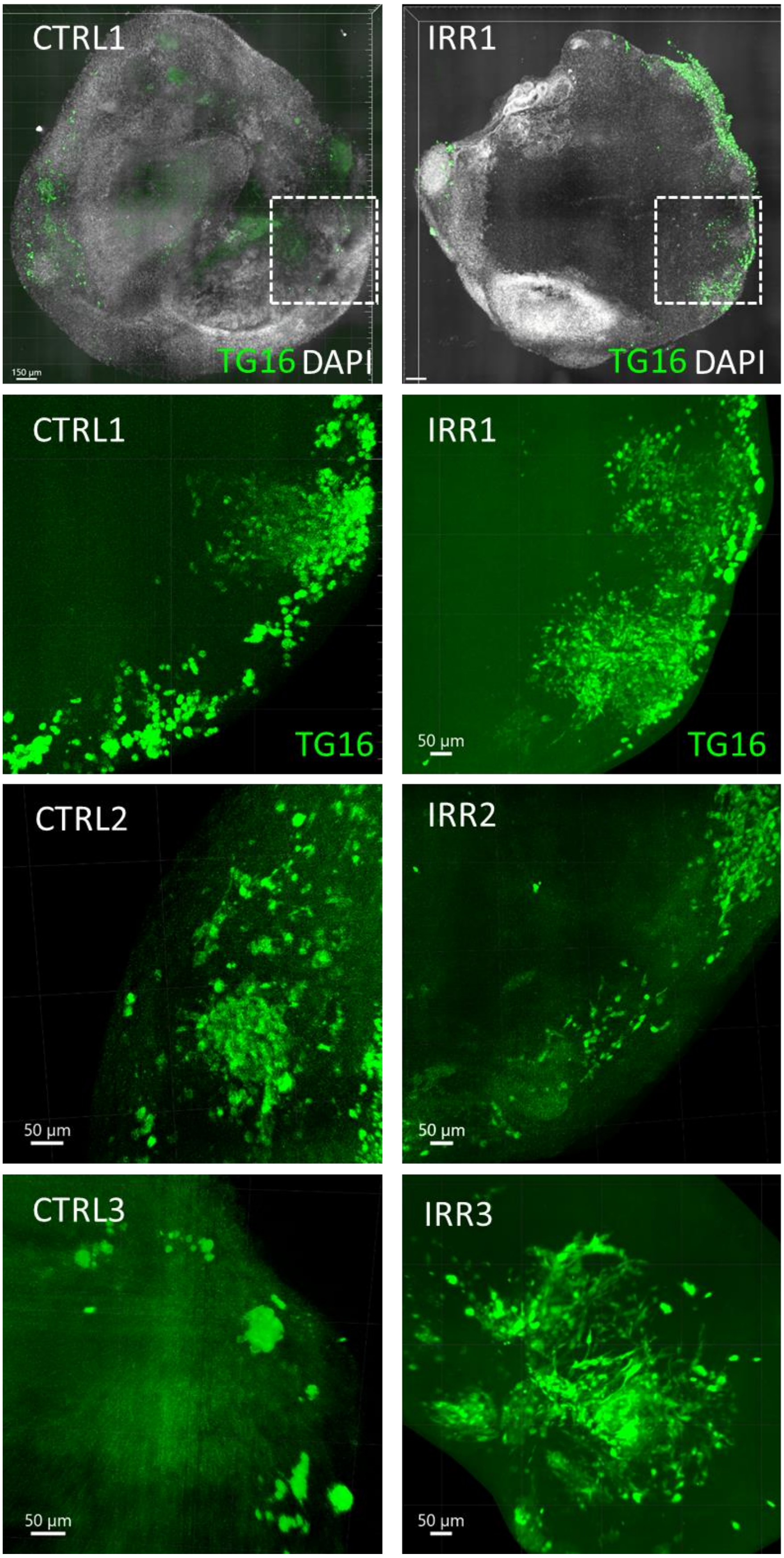

Fig. S10: TG16-GSCs co-cultured with CCOs acquired a mesenchymal-like morphology following irradiation. Related to Figure 7.

TG16 cells were co-cultured with CCOs obtained from GM25256 for two weeks before irradiation, then fixed two weeks later. 400 µm-thick sections were processed for immunofluorescence. TG16 cells exhibited a round morphology in unirradiated controls (CTRL1–3), whereas numerous cells adopted a mesenchymal-like morphology after irradiation (IRR1–3).

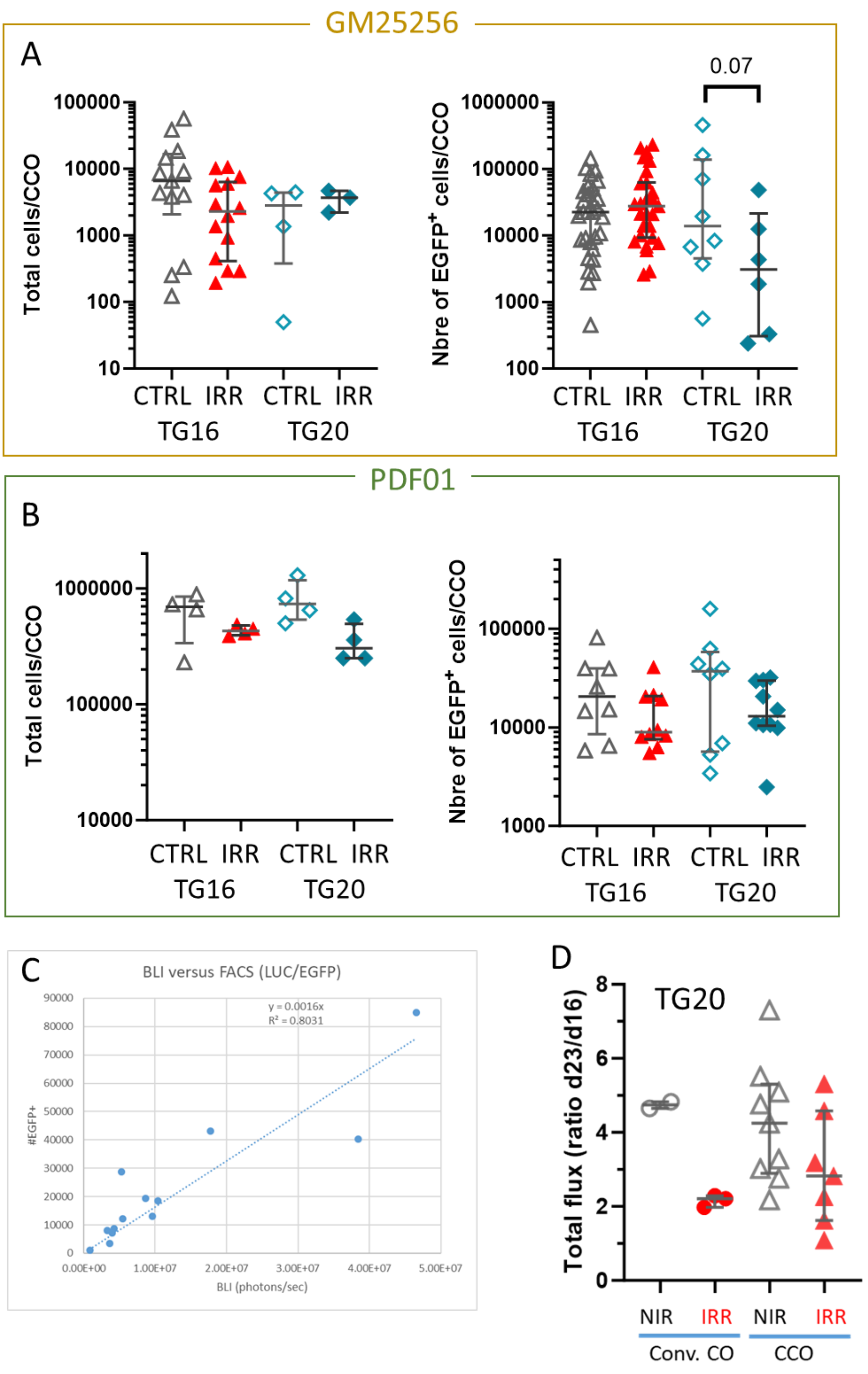

Fig. S11: Quantification of GSCs in coculture with CCOs after irradiation. Related to Figure 7.

TG16 and TG20 GSCs were co-cultured for two weeks with CCOs obtained from GM25256 (A and D) and PDF01 iPSC lines (B), then were irradiation and two weeks later processed by FACS. The total number of cells was determined with trucount calibration beads. (C) TG16-GSCs were co-cultured with CCOs, after which luciferase bioluminescence (photons/sec) was measured at day 28. The total number of EGFP-GSCs was then quantified by FACS the following day. (D) TG20 GSCs were co-cultured for two weeks with GM25256-derived COs and CCOs, then were irradiated (IRR), or not (NIR). The growth of TG20 cells was determined by Luciferase bioluminescence (photons/sec) 2 and 9 days later (D16 and D23). CCOs were derived from GM25256 iPSCs. Data are presented as median with interquartile range; each dot represents an individual CCO/CO. Comparisons across all groups were performed with the Kruskal–Wallis test with Dunn's post hoc multiple-comparisons test; no significant differences were detected.

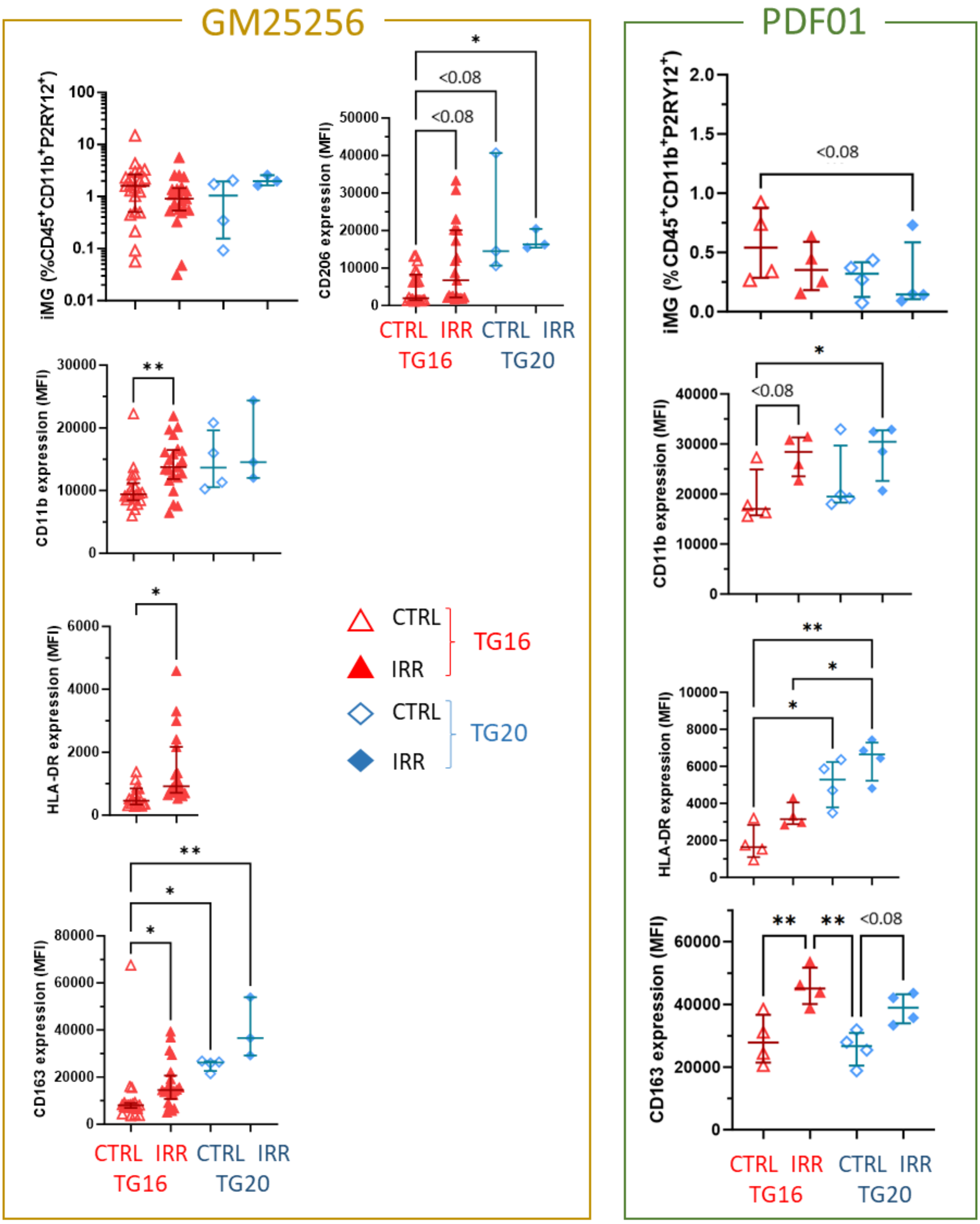

Fig. S12: Irradiation altered the phenotype of iMG in CCO cocultured with GSC lines. Related to Fig 7.

CCOs were cocultured with TG16 and TG20 GSCs for two weeks then irradiated at 2 Gy and analyzed by FACS 2 weeks later. The percentage of iMG ($CD45^+CD11b^+P2RY12^+$) was determined. The expression of CD11b, CD45, CD49d, HLA-DR, CD163 and CD206 was represented as the Mean Fluorescence Intensity (MFI). Data are presented as median with interquartile range; each dot represents an individual CCO/CO. Comparisons across all groups were performed with the Kruskal–Wallis test with Dunn's post hoc multiple-comparisons test (*: p<0.05, **: p<0.01).

Target	Species	Forward primer	Reverse primer
ATP5F1	Human	CTGTGCAGAACATGATGCGTCG	CTGTGCTTGAGCCTTCTTTGCC
18S	Human	GCAGAATCCACGCCAGTACAAG	TCCTCCTTCTGGAACTGCTGCA
AIF1	Human	CCCTCCAAACTGGAAGGCTTCA	CTTTAGCTCTAGGTGAGTCTTGG
ANXA1	Human	GCGAAACAATGCACAGCGTCAAC	CAACCTCCTCAAGGTGACCTGT
CCL20	Human	AAGTTGTCTGTGTGCGCAAATCC	CCATTCCAGAAAAGCCACAGTTTT
CCL2	Human	AGAGGCTGAGACTAACCCAGA	TTTCATGCTGGAGGCGAGAG
CCL8	Human	TATCCAGAGGCTGGAGAGCTAC	TGGAATCCCTGACCCATCTCTC
CCR2	Human	CAGGTGACAGAGACTCTTGGGA	GGCAATCCTACAGCCAAGAGCT
CD274	Human	TGCCGACTACAAGCGAATTACTG	CTGCTTGTCCAGATGACTTCGG
CD276	Human	CTGGCTTTCGTGTGCTGGAGAA	GCTGTCAGAGTGTTTCAGAGGC
CD44	Human	CCAGAAGGAACAGTGGTTTGGC	ACTGTCCTCTGGGCTTGGTGTT
CD70	Human	GCTGCAGCTGAATCACACAG	CTCTGGTCCATGCAGGAAGG
CD80	Human	CTCTTGGTGCTGGCTGGTCTTT	GCCAGTAGATGCGAGTTTGTGC
CSF1	Human	CCGGGGAAAGTGAAAGTTTGC	CAGACCAACAACAGCAGGGA
CSF1R	Human	GCTGCCTTACAACGAGAAGTGG	CATCCTCCTTGCCCAGACCAAA
CX3CR1	Human	CACAAAGGAGCAGGCATGGAAG	CAGGTTCTCTGTAGACACAAGGC
CX3CR1	Human	CTTACGATGGCACCCAGTGA	CAAGGCAGTCCAGGAGAGTT
CXCL10	Human	GGTGAGAAGAGATGTCTGAATCC	GTCCATCCTTGGAAGCACTGCA
CXCL12	Human	CTCCAGGTACTCCTGAATCCAC	CTCAACACTCCAAACTGTGCCC
CXCL16	Human	TACCTGTGGCACCTGACTCT	TGCCTACCATGTTGTCAGGG
CXCL2	Human	GGCAGAAAGCTTGTCTCAACCC	CTCCTTCAGGAACAGCCACCAA
CXCL2	Human	GGCAGAAAGCTTGTCTCAACCC	CTCCTTCAGGAACAGCCACCAA
ETV2	Human	AGTCGGACCGTGCCAGTTTGG	TGTTGCCAGTCCAACGGATGCA
FOSL2	Human	AAGAGGAGGAGAAGCGTCGCAT	GCTCAGCAATCTCCTTCTGCAG
IL34	Human	CCAAGGTGGAATCCGTGTTGTC	CACCTCACAGTCCTGCCAGTTT
KDR	Human	GGAACCTCACTATCCGCAGAGT	CCAAGTTCGTCTTTTCCTGGGC
MIF	Human	GCACCCAGACACCCTGAACCA	TGTGTCCAGGTCCTCCATGATG
MRC1	Human	AGCCAACACCAGCTCCTCAAGA	CAAAACGCTCGCGCATTGTCCA
MSR1	Human	TGCACAAGGCAGCTCACTTTGG	GTGCAAGTGACTCCAGCATCTTC
P2RY12	Human	TGCCAAACTGGGAACAGGACCA	TGGTGGTCTTCTGGTAGCGATC
PLIN2	Human	GATGGCAGAGAACGGTGTGAAG	CAGGCATAGGTATTGGCAACTGC
POSTN	Human	CAGCAAACCACCTTCACGGATC	TTAAGGAGGCGCTGATCCATGC
RUNX1	Human	CCACCTACCACAGAGCCATCAA	TTCACTGAGCCGCTCGGAAAAG
SPP1	Human	CGAGGTGATAGTGTGGTTTATGG	GCACCATTCAACTCCTCGCTTTC
TMEM119	Human	GGATAGTGGACTTCTTCCGCCA	GGAAGGACGATGGGTAATAGGC
VEGFA	Human	TTGCCTTGCTGCTCTACCTCCA	GATGGCAGTAGCTGCGCTGATA
VTCN1	Human	CTCACAGATGCTGGCACCTACA	GCAAGGTCTCTGAGCTGGCATT
GAPDH	Mouse	GACTCCACGACATACTCAGC	CCAGTATGACTCCACTCACG
MRC1	Mouse	GTTCACCTGGAGTGATGGTTCTC	AGGACATGCCAGGGTCACCTTT
MSR1	Mouse	CGCACGTTCAATGACAGCATCC	GCAAACACAAGGAGGTAGAGAGC
SPP1	Mouse	AGCAAGAAACTCTTCCAAGCAA	GTGAGATTCGTCAGATTCATCCG
TGFβ1	Mouse	AGCTGCGCTTGCAGAGATTA	AGCCCTGTATTCCGTCTCCT

Table S1: List of primers for RT-qPCR. Related to STAR Methods.
